# Lanthanide–carbamazepine complexes: synthesis, spectroscopic characterization, DFT Insights, molecular docking, and biological evaluation

**DOI:** 10.1038/s41598-026-35893-9

**Published:** 2026-02-11

**Authors:** Nora S. Mohamed, Mahmoud M. A. Mohamed, Mohamed R. Shehata, Ehab M. Abdalla

**Affiliations:** 1 Chemistry Department, Faculty of Science, New Valley University, Alkharga, 72511 Egypt; 2 Chemistry Department, Faculty of Science, Cairo University, Giza, 12613 Egypt

**Keywords:** Carbamazepine complexes, Lanthanide (III), DFT, Anti-cancer, Anti-microbial, Biochemistry, Cancer, Chemical biology, Chemistry, Computational biology and bioinformatics, Drug discovery

## Abstract

This work focuses on the synthesis of four novel lanthanide complexes: La (III), Ce (III), Nd (III), and Dy (III) with carbamazepine (CBZ). The geometric structural and bonding mode of synthesized complexes were accomplished by various analytical and spectroscopic techniques and the data reveal that the novel complexes have a 1:2 (Ln^3+^:CBZ) electrolytes with general formula [Ln(CBZ)_2_(H_2_O)Cl]Cl_2_.H_2_O and molecular weight: 753.83, 791.07, 777.19 and 777.43 for Ln^3+^ complexes respectively. The spectral studies investigated that CBZ interacts as a bidentate ligand via the amide group’s nitrogen and oxygen atoms in its moiety, and the geometric octahedral structure was confirmed by density function theory. On the other hand, PXRD data provided that the ligand and its complexes have nature that is crystalline. Antimicrobial activity was tested for their effectiveness against various Gram-positive and negative bacteria and anti-fungal efficacy, in addition to the efficiency of their cytotoxicity against cancer cell lines such as (Hep-G2 and MCF-7) to assess their potential as chemotherapy agents where the IC_50_ for Ln^3+^ complexes in the range (0.095–0.242) and (0.110–0.298) µM against Hep-G2 and MCF-7 cancer cell lines, respectively and La(III) demonstrated a high degree of activity, similarly, in the case of antimicrobials, it showed the highest activity. The ability of these complexes to bind to the active sites of the Bacillus subtilis receptor (PDB ID: 6A4M) and methionine adenosyl-transferases in liver tumors (PDB ID: 5A19) and human breast cancer MCF-7 (PDB ID: 4zvm) was evaluated using molecular docking studies utilizing MOA2022 software. According to the following pattern, the binding strength increased as the binding energy decreased: CBZ˂[Dy(CBZ**)**_2_(H_2_O)Cl]^2+^˂[Ce(CBZ**)**_2_(H_2_O)Cl]^2+^˂ [Nd(CBZ**)**_2_(H_2_O)Cl]^2+^˂[La(CBZ**)**_2_(H_2_O)Cl]^2+^, indicating that the highest binding affinity and possible biological activity were displayed by the La(III) complex.

**Supplementary Information:**

The online version contains supplementary material available at 10.1038/s41598-026-35893-9.

## Introduction

Carbamazepine (CBZ) is a pharmacological compound employed for the prophylaxis and treatment of seizures, commonly indicated for several epileptic illnesses, including bipolar mood disorders, trigeminal neuralgia, and both simple and complicated seizures^1^. The medicine absorbs only at a restricted rate and is essentially insoluble in water^2^. Being a pharmaceutically active substance and a non-steroidal anti-inflammatory medication, Carbamazepine is among the most prevalent medications in the environment^3,4^^5^. It is often used as a marker for human-caused contamination of surface water sources due to wastewater^5–7^^8^. CBZ, a tricyclic iminostilbene derivative, is a crucial and extensively used medicine for the treatment of epilepsy and some psychological disorders^4^. The interaction between drugs and different metals receives significant attention in the pharmaceutical sciences and medicinal chemistry. These reactions can significantly influence the efficacy, safety, and metabolism of drugs^9^. The ability of CBZ to form compounds with metal ions has been studied, which frequently have an octahedral shape. The interaction is often accomplished through bidentate coordination between the oxygen atom of the amide group and the nitrogen of the N-H bond^5^. The consequent complexes are applicable in various uses, such as electro catalysis, spectroscopy, biochemistry, and catalytic processes^10^. One of the most important aspects of the job that scientists do all around the world is using complicated chemicals in the chemical industry; the main goal has been to enhance the efficiency and selectivity of these processes in the last years^11^. Some lanthanide elements were selected for the following reasons: Complexes of lanthanides are one of the rapidly expanding subjects of inorganic chemistry research^12^. According to a review of published research, a large number of other recently synthesized substances or their derivatives form stable complexes^13,14^^15^, also, lanthanides occupy distinctive locations in the periodic table and display an intriguing variety in their characteristics. In recent years, extensive studies have shown that lanthanides are important in many biological processes, and they are useful tools for understanding the shapes and properties of biomolecules^16,17^. The research on lanthanides demonstrated their extensive range of applications, including therapies and medical diagnostics, agriculture, industry, and materials science^18^. Due to high coordination numbers, their structural motifs, fascinating magnetic, electronic, and spectroscopic properties, compounds containing lanthanide(III) ions have garnered a lot of attention in coordination chemistry recently^19^. The lanthanide (III) ion complexes have shown pharmacological properties such anti-inflammatory, anticoagulant, antiallergic, antibacterial^20^, and anticancer properties during the 1960s. In addition, Lanthanide (III) ion complexes were used to treat burns^21^..

The synthesis and application of a carbamazepine medication to characterize various lanthanide La(III), Ce(III), Nd(III), and Dy(III) complexes are the main topics of this investigation. Their anticancer and antibacterial properties were thoroughly assessed. For the rational design and evaluation of bioinorganic compounds, computational techniques like density functional theory (DFT) and molecular docking are becoming crucial in addition to experimental methods.

## Experimental

### Chemicals and instruments

At room temperature and in air, all of the synthetic work was carried out. Carbamazepine (CBZ) was supplied by UP Pharma, Assiut, Egypt. All chemicals used in this study, such as LaCl_3_**·**7H_2_O, CeCl_3_**·**7H_2_O, NdCl_3_**·**6H_2_O, and DyCl_3_**·**6H_2_O, as well as absolute ethanol, were analytical grade and purchased from Fluka and Aldrich Chemical, which were utilized without additional purification as received. The methods and procedures utilized for the application are contained in the supporting structure (Section S1).

### The metal complexes’ synthesis

The complexes were made by following a standard method (Scheme 2): equal amounts of ethanolic solutions of different metal salts (1 mmol = 0.371 g La(III); 0.372 g Ce(III); 0.358 g Nd(III); and 0.376 g Dy(III)) were mixed with an equal amount of an ethanolic solution of CBZ ligand (1 mmol, 0.236 g) in a 1:2 (Ln^3+^:CBZ) ratio, After that, the reaction mixture was refluxed at 70 °C for two hours while being constantly stirred. The formed solid complexes were filtered out and rinsed multiple times with a lot of pure EtOH to get rid of any leftover initial materials, and then they were dried in a vacuum at room temperature for 24 h^22,23^.

**Scheme 1:**
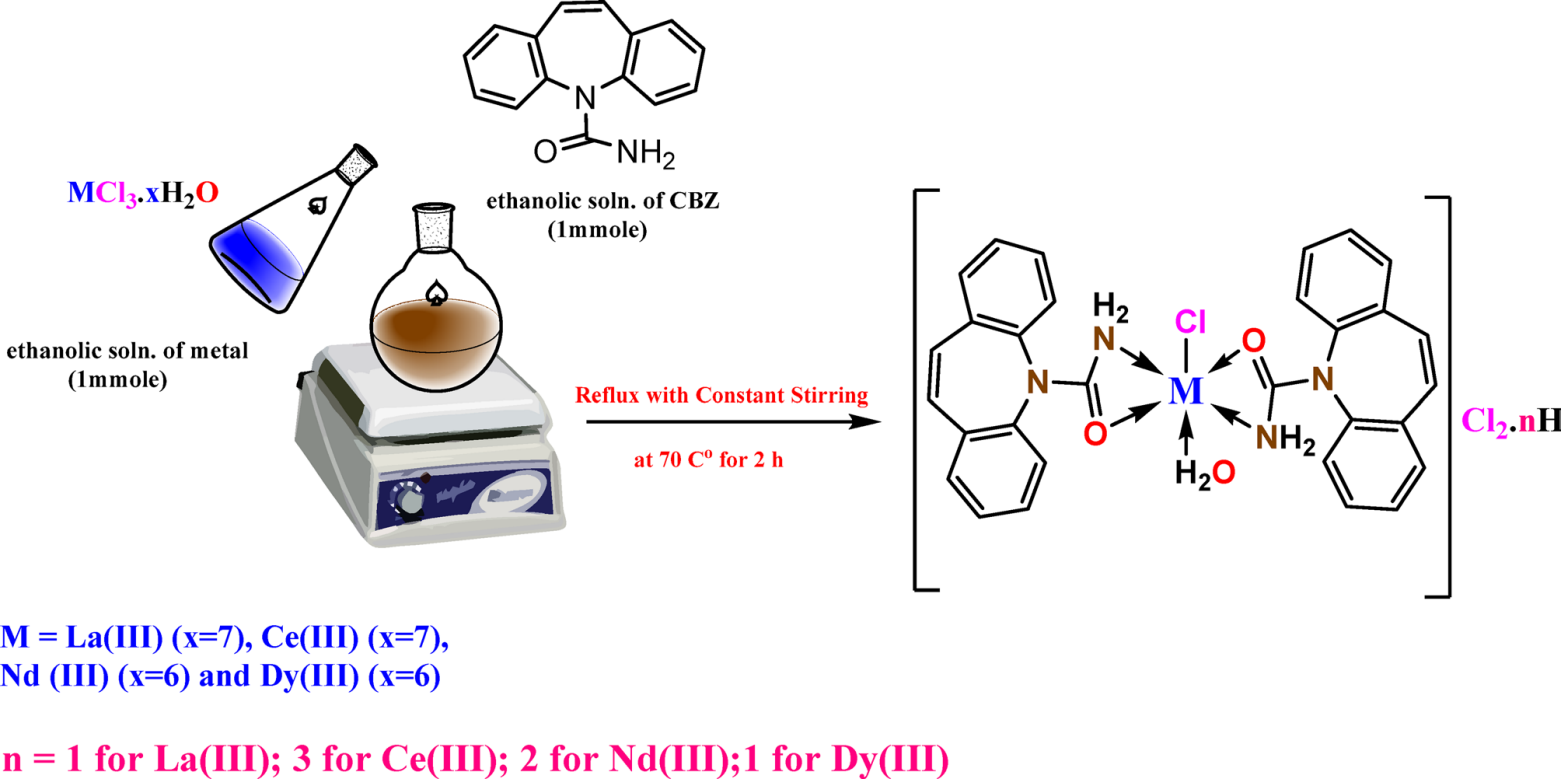
The suggested complexation reaction of metals La (III), Nd (III), Ce (III), and Dy (III) with CBZ.

### Computational study

The B3LYP functional was used in Density Functional Theory (DFT) simulations to identify the lowest energy equilibrium geometries of the CBZ ligand and its metal complexes. In the DFT, the lanthanides metal atoms in these calculations were based on the SDD basis set, whereas the C, H, N, O, and Cl atoms were based on the 6–311 + + G(d, p) basis set^24^. The software package Gaussian 09 was utilized to perform these computations.

### Antimicrobial bioassay

Using the method of modified well diffusion, we tested the antimicrobial effectiveness of CBZ and its synthesized complexes against fungi (*Aspergillus flavus* and *Candida albicans)*, a type of yeast; Gram-positive bacteria (*Bacillus subtilis* and *Staphylococcus aureus)*; and Gram-negative bacteria (*Proteus vulgaris* and *Escherichia coli)*. The standard antibacterial agent is gentamicin, and the typical antifungal agent is ketoconazole. (Section S2)^25,26^^27^,

### Anticancer activity

**-** *Mammalian cell lines*: HepG2 cells (human liver cancer cell line) and MCF-7 cells (human breast cancer cell line) were obtained from the American Type Culture Collection (ATCC, Rockville, MD).
**-** *Chemicals Used*: MTT and trypan blue dye were purchased from Sigma (St. Louis, Mo., USA).

Fetal Bovine serum, RPMI-1640, HEPES buffer solution, L-glutamine, gentamycin, and 0.25% Trypsin-EDTA were purchased from Lonza (Belgium).

**-** *Cell line Propagation*: The cells were grown on RPMI-1640 medium supplemented with 10% inactivated fetal calf serum and 50 µg/ml gentamycin. The cells were maintained at 37 °C in a humidified atmosphere with 5% CO_2_ and were subcultured two to three times a week.
**-** *Cytotoxicity evaluation using viability assay*: To assess the cytotoxicity activity of CBZ and its complexes against breast and liver cancer cell lines, tumor cell lines were planted in 96-well plates at a density of 1 × 10^5^ cells/well and treated for 24 h. In triplicate, test chemicals in a range of concentrations (including 12 concentrations for cisplatin) were added after being dissolved in PBS. The MTT assay was employed to assess cell vitality following an additional 24-hour incubation. DMSO was used to dissolve the formazan crystals following the addition of the MTT solution and a four-hour incubation period. Absorbance at 590 nm was used to ascertain the viability of the cells. Plotting survival curves with GraphPad Prism software allowed for the determination of the IC_50_ values (Section S3)^28,29^.

### Docking molecules

Using the MOA2022 software^30^ molecular docking studies were conducted to investigate possible binding mechanisms of the methionine adenosyl transferase in human breast cancer MCF-7 (PDB ID: 4ZVM) and the Bacillus subtilis receptor site (PDB ID: 6A4M) in liver malignancies (PDB ID: 5A19)^31,32^. The Protein Data Bank was used to get experimental crystallographic structures of the protein. Before docking, the protein was prepared by removing water molecules bound to DNA base pairs and replacing them with hydrogen. Many docking simulations were run using default parameters, and conformations were selected based on a combination of S score data, E conformation, and proper fitting with the relevant amino acids in the binding pocket; the target protein was kept rigid (the MOE docking program automatically prepare the protein and the site finder produce the best cavity appropriate for docking). Ligands (our compounds in this study) were obtained from the output of optimized DFT data.

## Results and discussion

### Characteristics

A variety of analytical techniques, including FTIR, UV-Vis, mass, PXRD, elemental analysis, and thermogravimetric studies, were used to describe the produced complexes. The chemical analytical data showed that the formation of complexes with the stoichiometry of 1:2 (Ln^3+^: CBZ); theses complexes are electrolytic in nature where the molar conductivity value of complexes between 201 and 223 Ω^−1^cm^2^mol^−1^ in 10^− 3^ M DMF solution^33^ Table 1, colored, stable in air, non-hygroscopic and were soluble in organic solvents as (DMF, DMSO) but insoluble in water, partially soluble in ethanol.

**Table 1:** Micro analytical data of CBZ and its lanthanide complexes.

Compound	Mol. formula	Mol. Wt.	Color, yield (%)	Conductivity ^Ʌ^m	m.*p*. ^o^C	Calculated (Found)			
						C	H	*N*	M
CBZ	C_15_H_12_N_2_O	236.27	White	-	192	76.25 (76.78)	5.12 (5.18)	11.86 (12.00)	-
[La(CBZ)_2_(H_2_O)Cl]Cl_2_.H_2_O	C_30_H_28_LaCl_3_N_4_O_4_	753.83	Off white 88	223	> 300	47.80 (47.59)	3.74 (3.51)	7.43 (6.7)	18.43 (18.87)
[Ce(CBZ)_2_(H_2_O)Cl]Cl_2_.3H_2_O	C_30_H_32_CeCl_3_N_4_O_6_	791.07	Beige 77.6	213	> 300	45.55 (45.34)	4.08 (4.02)	7.08 (6.88)	17.71 (17.12)
[Nd(CBZ)_2_(H_2_O)Cl]Cl_2_.2H_2_O	C_30_H_30_NdCl_3_N_4_O_5_	777.19	Pale pink 73	211	> 300	46.36 (46.13)	3.89 (3.75)	7.21 (6.85)	18.56 (18.23)
[Dy(CBZ)_2_(H_2_O)Cl]Cl_2_.H_2_O	C_30_H_28_DyCl_3_N_4_O_4_	777.43	Yellow 91.7	201	> 300	46.35 (47.09)	3.63 (3.86)	7.21 (7.62)	20.90 (20.35)

Where: ^**Ʌ**^**m** represents the molar conductivity, measured in ohm^−¹^ cm^²^ mol^− 1^.

### ^*1*^*H and*^13^*C-NMR spectra*

**Fig. 1:**
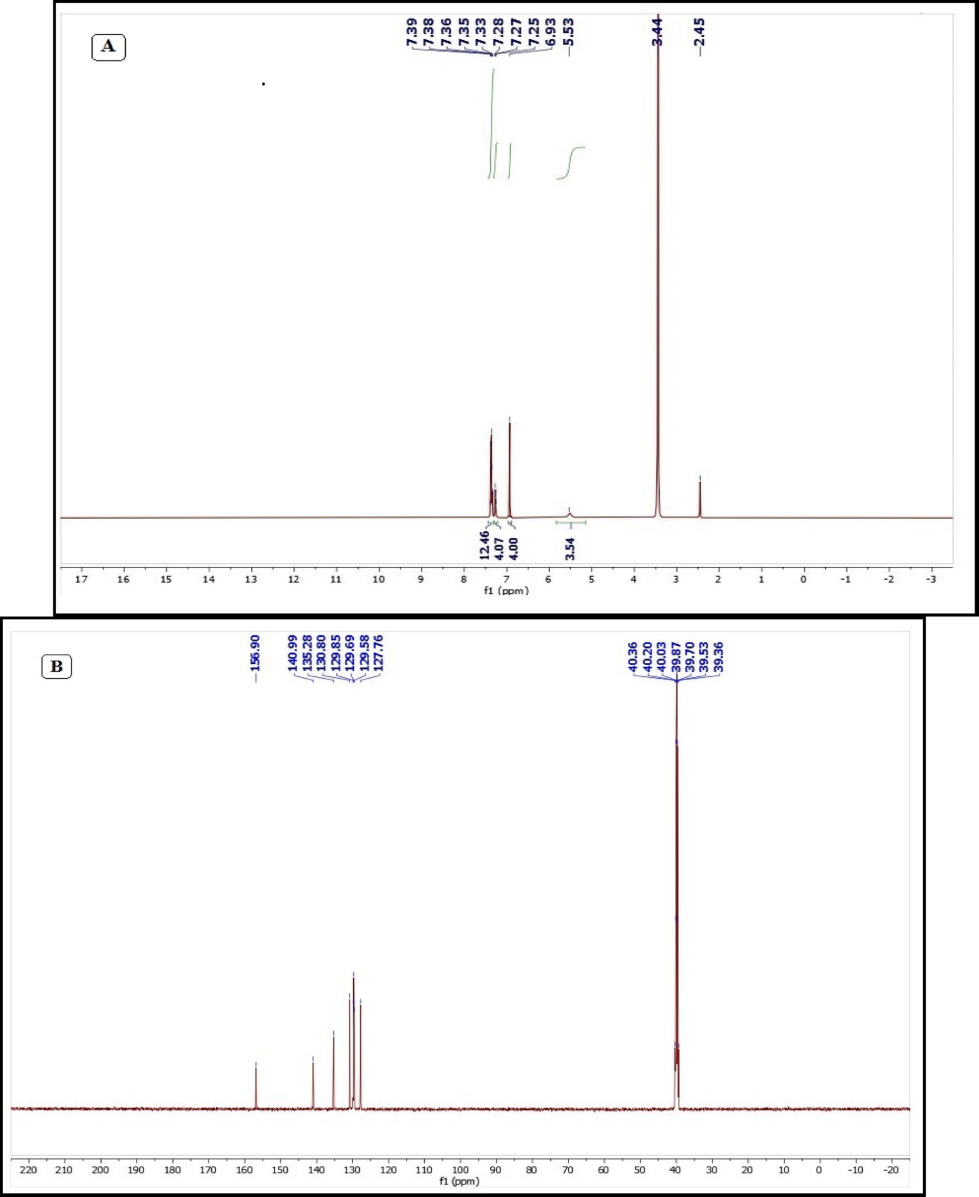
^1^H-NMR (**A**) and^13^C-NMR (**B**) spectrum of La(III) CBZ complex in DMSO d_6_.

The^1 ^H-NMR spectrum of the La(III) CBZ complex was acquired in DMSO-d_6_. A signal corresponding to the methine (CH) proton was observed at δ 5.53 ppm. A singlet at δ 6.93 ppm was assigned to the two protons of the NH_2_ group. Multiple signals in the aromatic region (δ 7.25–7.39 ppm) are attributed to the aromatic ring protons (Fig. 1 A). The ^13^C-NMR spectrum of the La(III) CBZ complex in DMSO-d_6_ (Fig. 1B) displayed signals at δ 127.76, 129.58, 129.69, 129.85, 130.80, 135.28, and 140.99 ppm, corresponding to the aromatic carbons. An additional signal at δ 156.90 ppm was assigned to the carbonyl (C = O) carbon.

### FTIR spectra

**Fig. 2:**
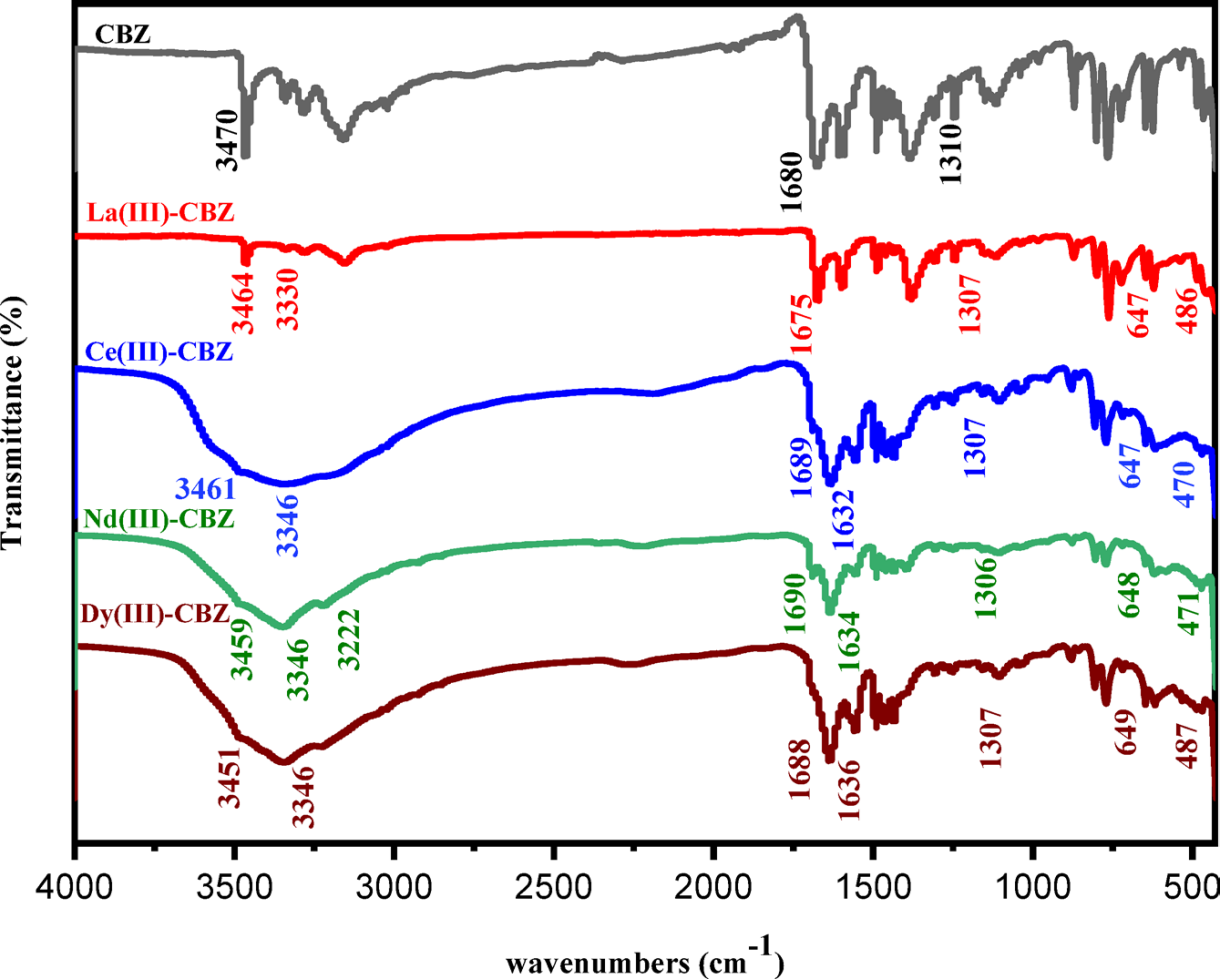
FT-IR spectra (cm^− 1^) of the CBZ ligand and its lanthanide complexes in the region of 4000–400 cm^− 1^.

To assess how CBZ binds to the metal ion in the complexes, characteristic FT-IR bands were investigated within the range from 4000 to 400 cm^− 1^. The significant infrared absorption frequencies of lanthanide (III)-CBZ complexes have been displayed in Fig. 2 and given in Table 2. The spectrum of pure CBZ showed a sharp peak at 3470 cm^− 1^ corresponding to –NH_2_^2,34^, as well as an absorption band at 1680 cm^− 1^ and 1310 cm^− 1^ due to C = O of the amide group and C-N, respectively^5,35^. On complexation, the sharp peaks of the CBZ ligand at 3470 cm^− 1^ shift to lower frequencies in Complexes at 3464, 3461, 3459 and 3451 cm^− 1^ for Ln^3+^ complexes, respectively this confirm the nitrogen in the amino group coordinated with metal ions, as well as the peak related to the C = O of amide group shifted to higher and lower frequencies in all complexes (1675, 1689, 1690 and 1688 cm^− 1^) for La(III), Ce(III), Nd(III) and Dy(III) complex, respectively, which confirm the participation of the amide moiety’s C = O in the complexation reaction^36,37^. All complex spectra showed a broad band at (3330–3347) and (954–980) cm^− 1^, attributed to the ν(OH)/H_2_O confirms the presence of hydrated and coordinated water molecules^38^. New bands appeared in all complexes in the range of (647–649) and (470–487) cm^− 1^, which are attributed to ν(M–O) and ν(M–N), respectively^39–41^^42^,. Consequently, it is presumed that the CBZ ligand acts as a bidentate ligand with NO metal ion coordination sites based on the results of FT-IR spectra.

**Table 2:** FT-IR spectra of the ligand CBZ and its compounds with lanthanides.

Compound	v(OH)/H_2_O	v(NH_2_) _1_^o^_amine_	v(C = O)_amide_	v(C-*N*)_alph_.	v(M-O)	v(M-*N*)
CBZ	—	3470	1680	1310	—	—
[La(CBZ)_2_(H_2_O)Cl]Cl_2_.H_2_O	3330	3464	1675	1307	647	486
[Ce(CBZ)_2_(H_2_O)Cl]Cl_2_.3H_2_O	3346	3461	1689	1307	647	470
[Nd(CBZ)_2_(H_2_O)Cl]Cl_2_.2H_2_O	3347	3459	1690	1306	648	472
[Dy(CBZ)_2_(H_2_O)Cl]Cl_2_.H_2_O	3346	3451	1688	1310	649	487

### Mass spectra

**Fig. 3:**
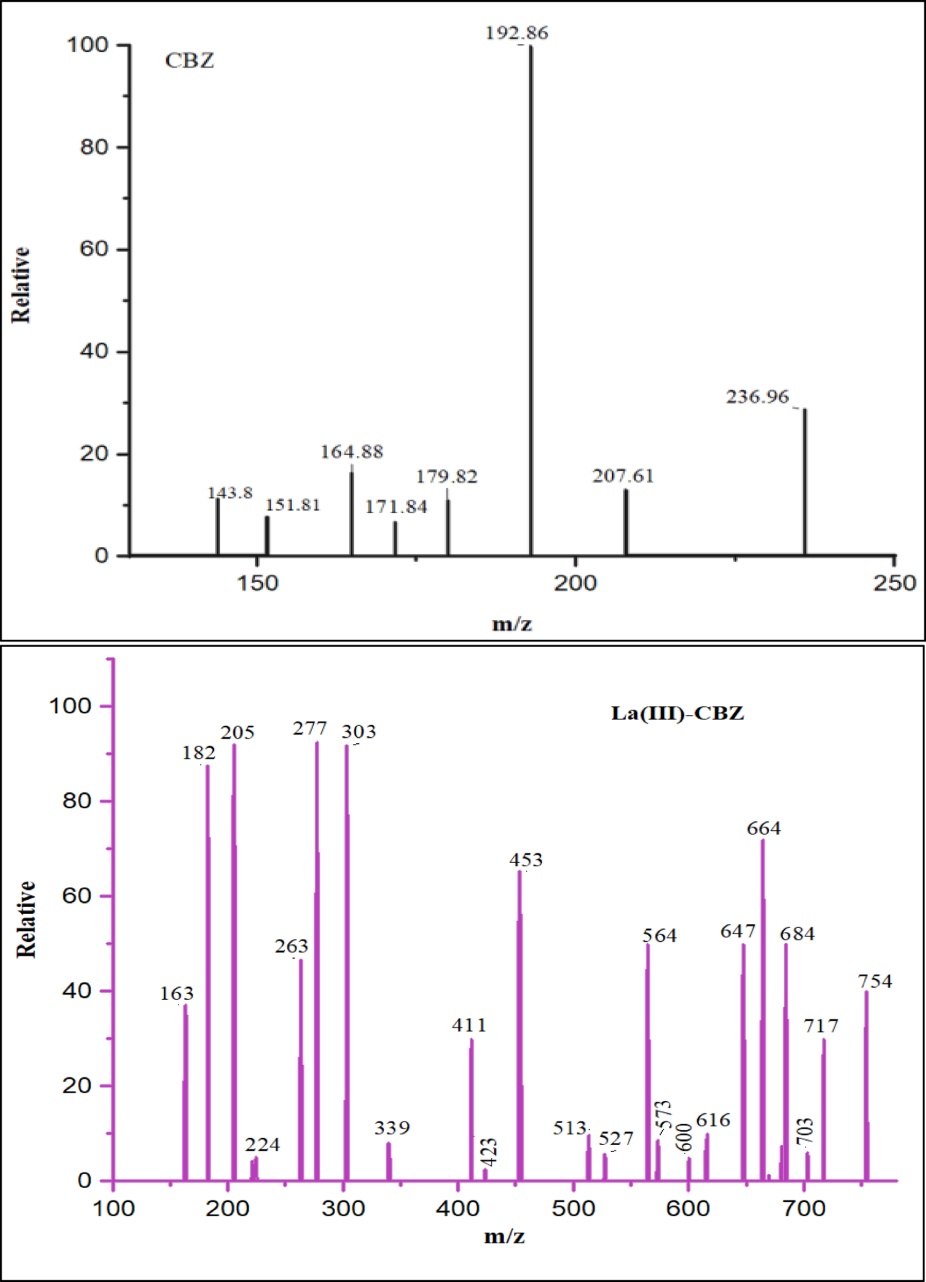
Mass spectra diagram of CBZ ligand and complex La(III)-CBZ.

**Scheme 2:**
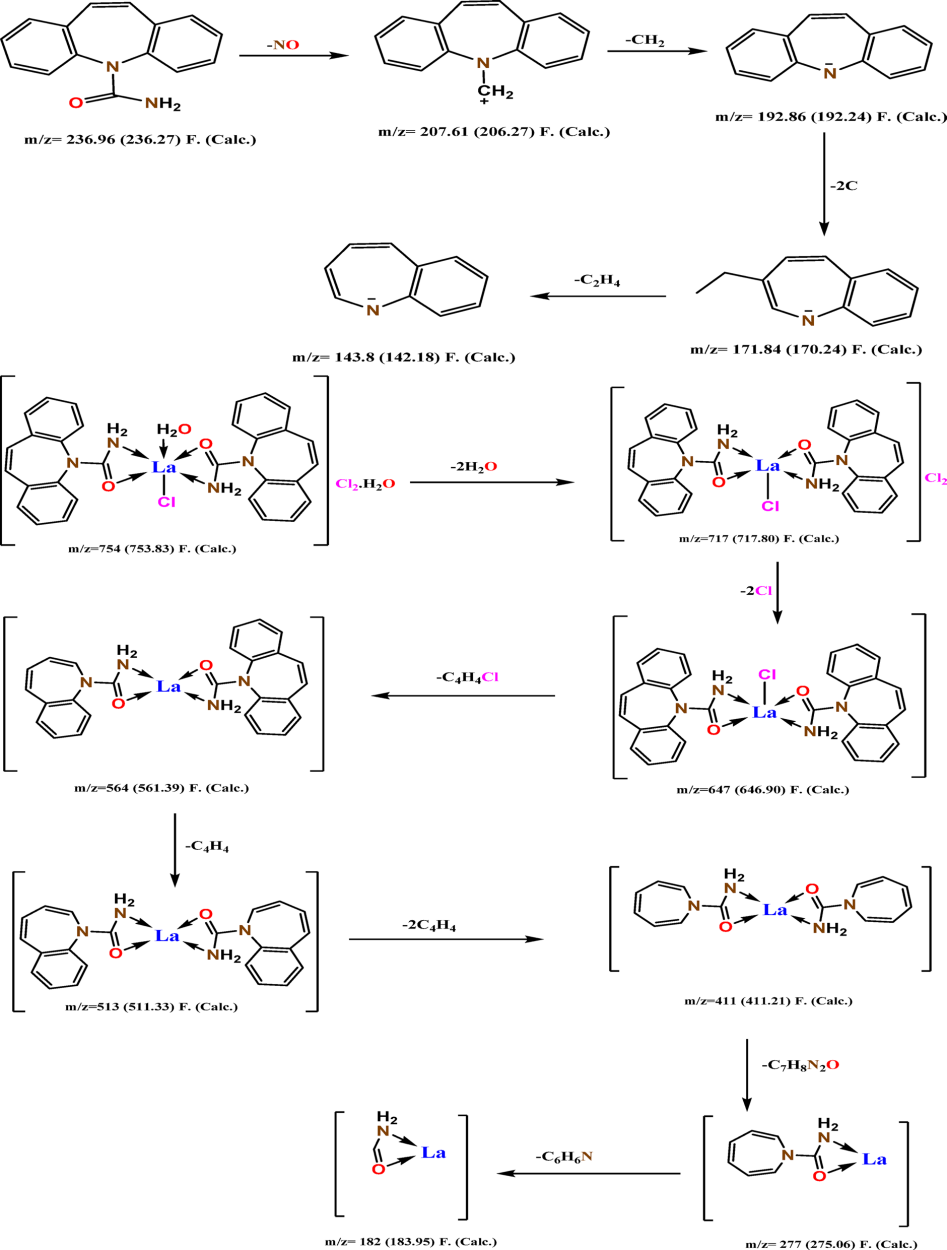
Fragmentation pattern of CBZ ligand and complex La(III)-CBZ.

Mass spectrometry is an effective method that may be utilized for the purpose of determining unknowns, investigating the structure of molecules, and gaining insight into the fundamental principles of chemistry. We conducted mass spectrum analyses of representative complexes to validate the formula weights suggested based on elemental analyses. Fig.s 3. and fig.s S1 displays the mass spectra diagram of the ligand (CBZ) and its synthesized complexes. The molecular formula of the ligand (CBZ) (C_15_H_12_N_2_O) (M.wt. 236.27) is indicated from its mass spectrum (Fig. 3). The observed peak at m/z = 236.96 (molecular ion peak), related to C_15_H_12_N_2_O, atomic mass *m/z* = 236.27, the spectrum shows important fragment ions in the range m/z = 207.61 for C_15_H_12_N^+^, m/z = 192.86 for C_14_H_10_N^−^,m/z = 171.84 for C_12_H_12_N^−^ and m/z = 143.8 for C_10_H_8_N^−^ (Scheme 2). The spectrum of complex La(III)-CBZ (C_30_H_28_LaN_4_O_4_Cl_3_, atomic mass = 753.83), shows molecular ion peak [M^1+^] at m/z = 754 and a base peak at m/z = 277 for [C_7_H_8_LaN_2_O]. Additionally, the mass spectra displayed a multi-peak pattern that included a variety of fragment ions within the range of m/z = 717 for [C_30_H_24_LaN_4_O_2_Cl_3_], 647 for [C_30_H_24_LaN_4_O_2_Cl], 564 for [C_26_H_22_LaN_4_O_2_], 513 for [C_22_H_20_LaN_4_O_2_], 411 for [C_14_H_16_LaN_4_O_2_], 277 for [C_7_H_8_LaN_2_O] and 182 for [CH_3_LaNO], (Scheme 2**)**. These peaks were exactly in line with the metal complex’ proposed chemical formula. The spectrum of complex Ce(III)-CBZ (C_30_H_32_CeN_4_O_6_Cl_3_, atomic mass = 791.07), show molecular ion peak [M^1+^] at m/z = 792, the molecular ion undergoes fragmentations at m/z = 770.53 for [C_30_H_30_CeN_4_O_5_Cl_3_], m/z = 735.6 for [C_30_H_26_CeN_4_O_3_Cl_3_], m/z = 720.22 for [C_30_H_24_CeN_4_O_2_Cl_3_], m/z = 616 for [C_30_H_24_CeN_4_O_2_], m/z = 560.57 for [C_26_H_22_CeN_4_O_2_], m/z = 247.23 for [C_2_H_10_CeN_4_O] and m/z = 230.43 for [C_2_H_9_CeN_3_O], these fragmentation patterns were perfectly match with the predicted molecular structure of the Ce(III) complex. The mass spectrum of complex Nd(III)-CBZ (C_30_H_30_NdN_4_O_5_Cl_3_, atomic mass = 777.19), exhibited a peak assigned to the molecular ion peak at m/z = 777.26 for [C_30_H_30_NdN_4_O_5_Cl_3_] and a base peak at m/z = 588.43 for [C_22_H_20_NdN_4_O_2_Cl_2_]. The pattern of several peaks in the mass spectrum reveals peaks for fragment ions in the range of m/z = 687.91 for [C_30_H_24_NdN_4_O_2_Cl_2_], 588.43 for [C_22_H_20_NdN_4_O_2_Cl_2_], 503.97 for [C_18_H_18_NdN_4_O_2_Cl], 449.04 for [C_14_H_16_NdN_4_O_2_Cl], 298.8 for [C_2_H_8_NdN_4_O_2_Cl], 190.42 for [CH_6_NdN_2_] and 159.46 for [NdNH_3_]. These data match with its suggested molecular formula. On the other hand, the molecular ion peak of complex Dy(III)-CBZ (C_30_H_28_DyN_4_O_4_Cl_3_, atomic mass = 777.43) was observed at m/z = 777.67 and a base peak at m/z = 192.78 for [CH_2_DyO] which is acceptable with its proposed molecular structure, this molecular ion undergoes fragmentation in the range of m/z = 759.83 for [C_30_H_26_DyN_4_O_3_Cl_3_], 741.2 for [C_30_H_24_DyN_4_O_2_Cl_3_], 691.4 for [C_26_H_22_DyN_4_O_2_Cl_3_], 504.3 for [C_14_H_16_DyN_4_O_2_Cl_2_], 429.16 for [C_8_H_12_DyN_4_O_2_Cl_2_], 318.57 for [C_2_H_8_DyN_4_O_2_Cl], 270.87 for [C_2_H_10_DyN_4_O] and 192.78 for [CH_2_DyO]. The proposed fragmentation pattern of Ce(III), Nd(III), and Dy(III) complexes is discussed in (Scheme S1). All the mass data are in agreement with the fragmentation pattern of the suggested complexes.

### Properties of magnetic and electronic transitions

The electronic absorption spectra in DMF solution at room temperature were demonstrated from 200 to 800 nm in order to provide more relevant information on the UV-visible spectral properties of ligand (CBZ) and its metal complexes (Fig. S2). As shown in Table 3, the absorption of CBZ can be noticed at wavelengths of 295, 320, and 370 nm. It is possible to credit the first band at 295 nm to transitions of π–π*, whereas the second two bands at 320 and 370 nm can be attributed to n–π* transitions in the case of unsaturated hydrocarbons^5^. In contrast, the metal chelate’s electronic spectra demonstrate π →π* and n →π* transitions of the ligand, which are displaced to higher values upon complexation, indicating successful chelation. No spectra of the complexes exhibited bands in the visible spectrum. This is likely attributable to the weakness of the f–f bands, which are hidden by the powerful charge transfer bands^43^. Only the La(III) complex is diamagnetic, as indicated by the magnetic moments of the lanthanide (III) complexes. The other lanthanide ion complexes are paramagnetic^44^.

**Table 3:** The data of electronic spectra in DMF solution of CBZ ligand and lanthanide complexes.

No.	Compound	Wavelength λ_max_ (nm)	µ_eff_ BM	Molar absorptivity (ε) M⁻¹∙cm⁻¹
CBZ	C_15_H_12_N_2_O	295, 320, 370	—	(1.65, 1.12, 0.76) ×10^3^
[La(CBZ)_2_(H_2_O)Cl]Cl_2_·H_2_O	C_30_H_28_DyCl_3_N_4_O_4_	293, 338, 376	Dia	(1.99, 1.38, 0.74) ×10^3^
[Ce(CBZ)_2_(H_2_O)Cl]Cl_2_·3H_2_O	C_30_H_28_LaCl_3_N_4_O_4_	296, 335, 376	2.54	(1.86, 1.23, 0.68) ×10^3^
[Nd(CBZ)_2_(H_2_O)Cl]Cl_2_·2H_2_O	C_30_H_30_NdCl_3_N_4_O_5_	295, 348, 378	3.62	(1.74, 1.03, 0.68) ×10^3^
[Dy(CBZ)_2_(H_2_O)Cl]Cl_2_·H_2_O	C_30_H_32_CeCl_3_N_4_O_6_	295, 340, 378	10.65	(1.78, 1.00, 0.66) ×10^3^

### PXRD analysis

**Fig. 4:**
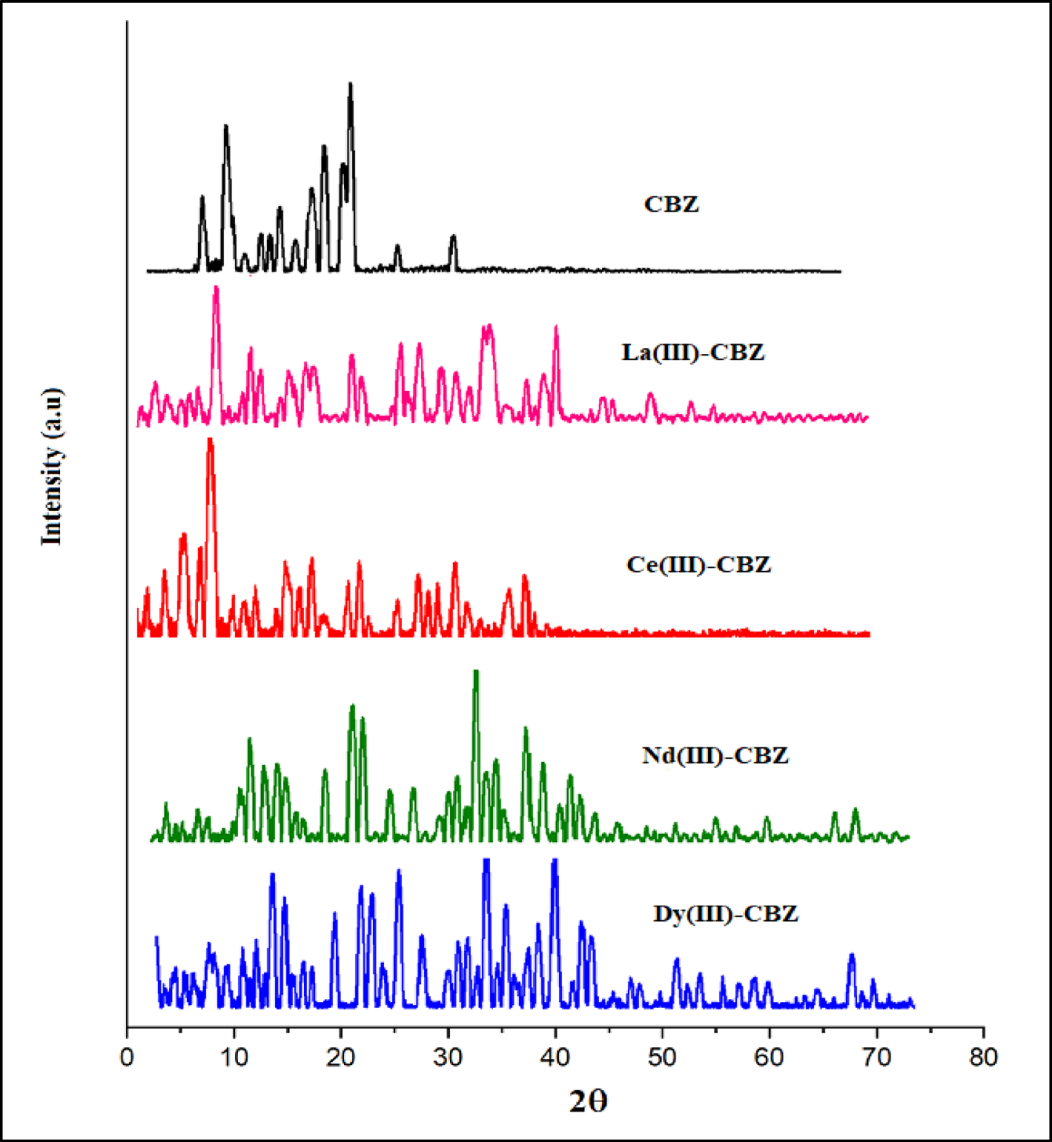
X-ray powder diffraction pattern of CBZ-ligand and lanthanide compounds.

To gain more information related to the metal complexes’ structure, X-ray diffraction was employed. The diffractograms acquired for CBZ ligand and lanthanide complexes displayed in Fig. 4. By comparing the ligand and complexes’ diffractograms, it is evident that the ligand’s powder X-ray diffraction pattern differs entirely from that of its metal complexes; the crystalline nature of the complexes was detected. According to literature, this behavior may be a result of the presence of water molecules have been incorporated into the coordination sphere^45^. By using Debye Scherrer Eqs. 4^6,47^ (D = K*λ)/β*Cosϴ); where D is crystal size (nm), K is a Scherrer’s constant (0.95), λ is a wavelength of x-ray source (0.15406 nm), β is the width at half maximum of the diffraction peak by radians unit (FWHM), ϴ is the angle of diffraction or the peak position measured in radians^48^, we calculated the complexes’ crystallite size (D), which the estimated average crystallite size is 12.38, 10.13, 10.8, 14.01 and 1.97 nm for the ligand CBZ, the complexes of La(III)-CBZ, Ce(III)-CBZ, Nd(III)-CBZ and Dy(III)-CBZ, respectively. PXRD data of CBZ and lanthanide complexes are shown in Table 4.

Indexing of PXRD peaks using Miller indices (hkl) confirms the crystalline nature of CBZ and reveals clear structural changes upon coordination with La(III), Ce(III), Nd(III), and Dy(III) ions. The observed variations in (hkl) values indicate lattice rearrangement and partial distortion, providing strong evidence for successful complex formation.

**Table 4:** PXRD results for CBZ ligand and lanthanide complexes.

Compound	Angle 2θ	d-spacing nm	FWHM nm	hkl	Crystallite size (D) nm
CBZ	12.91707^o^	0.68480768	0.50013	100	15.98590227
	15.2679 ^o^	0.57985437	0.75019	110	10.68439846
	24.84688 ^o^	0.35805446	0.5094	200	15.96931677
	27.20162 ^o^	0.32756999	1.18193	210	6.915378019
La(III)-CBZ	13.06668 ^o^	0.67700025	0.62303	100	12.83440272
	33.09849 ^o^	0.27043316	3.09388	211	2.678707837
	40.81304 ^o^	0.22091972	1.28645	300	6.588859318
	47.83521 ^o^	0.18999933	0.47166	320	18.42545832
Ce(III)-CBZ	10.33483 ^o^	0.85593780	0.63877	100	12.48760602
	13.0746 ^o^	0.67686987	0.70735	110	11.30456103
	20.77844 ^o^	0.42739114	44.04217	200	0.183385678
	37.72753 ^o^	0.23810527	0.43655	320	19.23077481
Nd(III)-CBZ	14.735 ^o^	0.60070309	0.46889	100	17.08379915
	25.29237 ^o^	0.35184788	1.63438	111	4.981585841
	37.01814 ^o^	0.24264874	0.48127	211	17.40735034
	42.00859 ^o^	0.21490471	0.51364	220	16.56749921
Dy(III)-CBZ	16.76595 ^o^	0.52836609	2.67178	100	3.005518746
	26.51073 ^o^	0.33594786	5.65418	111	1.44348706
	37.50108 ^o^	0.23963411	4.34385	210	1.931361944
	44.3026 ^o^	0.20429452	5.61713	221	1.52700273

### Scanning (SEM) and transmission (TEM) electron microscope

**Fig. 5:**
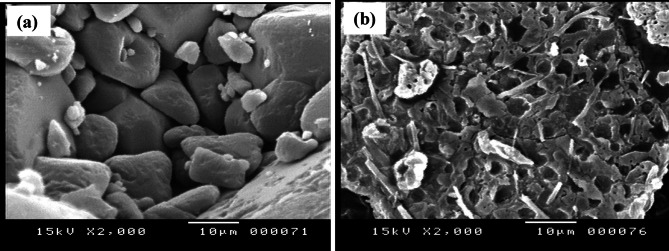
SEM of (**a**): CBZ; and (**b**): La(III)-CBZ complex.

**Fig. 6:**
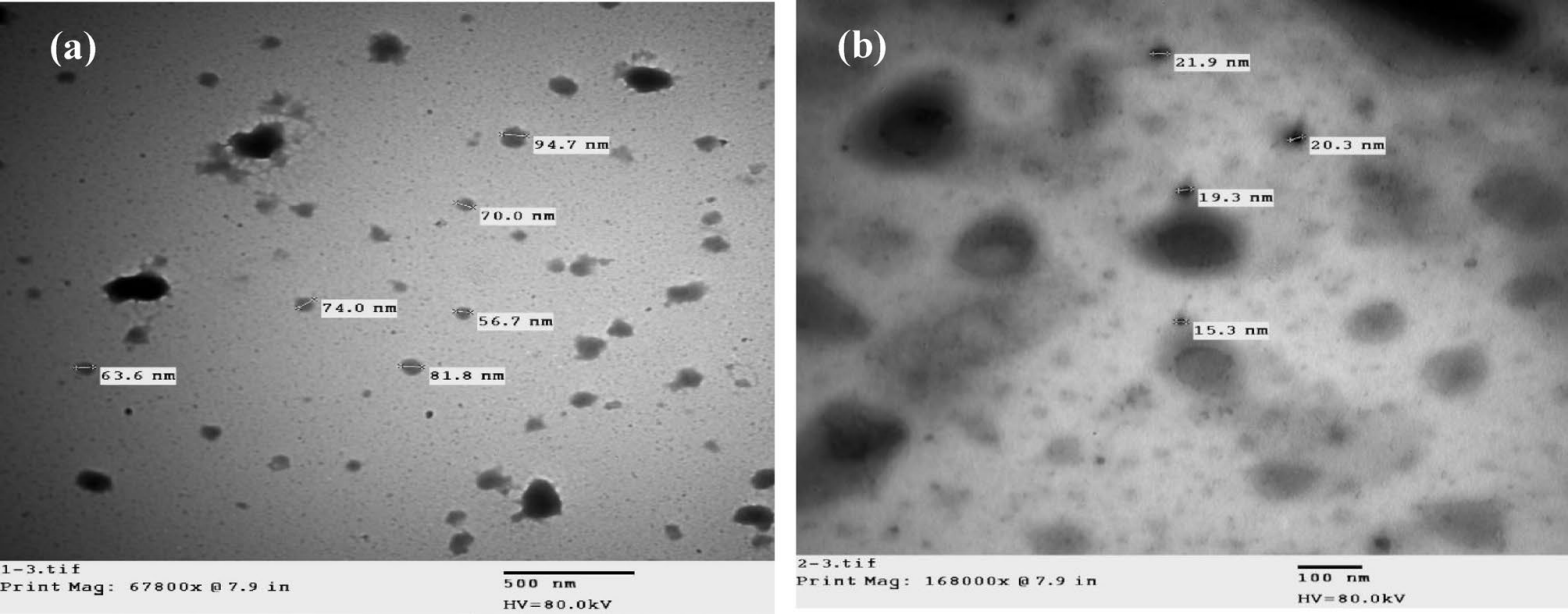
TEM images of (**a**): CBZ; and (**b**): La(III)-CBZ complex.

The surface morphology and particle size of CBZ and La(III)-CBZ complex was analyzed by Scanning electron microscopy (SEM) at an accelerating voltage of 15 kV, a working distance of 10 μm and Transmission electron microscopy (TEM). The SEM images reveal significant differences between (a) carbamazepine (CBZ) and (b) its La(III) complex, as illustrated in Fig. 5; CBZ exhibits irregular, aggregated particles characterized by rough surfaces and poorly defined grain boundaries, signifying low crystallinity and weak intermolecular packing. Conversely, the La–CBZ complex displays compact, densely arranged particles with distinct forms and smoother surfaces, indicating improved structural order and heightened stability. This morphological shift offers compelling evidence for complex formation, as metal coordination markedly modifies the surface roughness and packing behavior. The TEM study of the CBZ ligand and La–CBZ complex present in the Fig. 6(a, b) where the La(III) CBZ shows that nanoscale particles with a shape that is almost spherical and little aggregation form, which the particles varied in size from 15.3 to 21.9 nm. Areas with deeper contrast are attributed to La-CBZ domains while lighter areas represent the surrounding matrix. The findings confirm the creation of homogeneous La–CBZ nanoparticles consistent with SEM and XRD data, affirming their nanocrystalline nature and successful synthesis.

### Thermal analysis

**Fig. 7:**
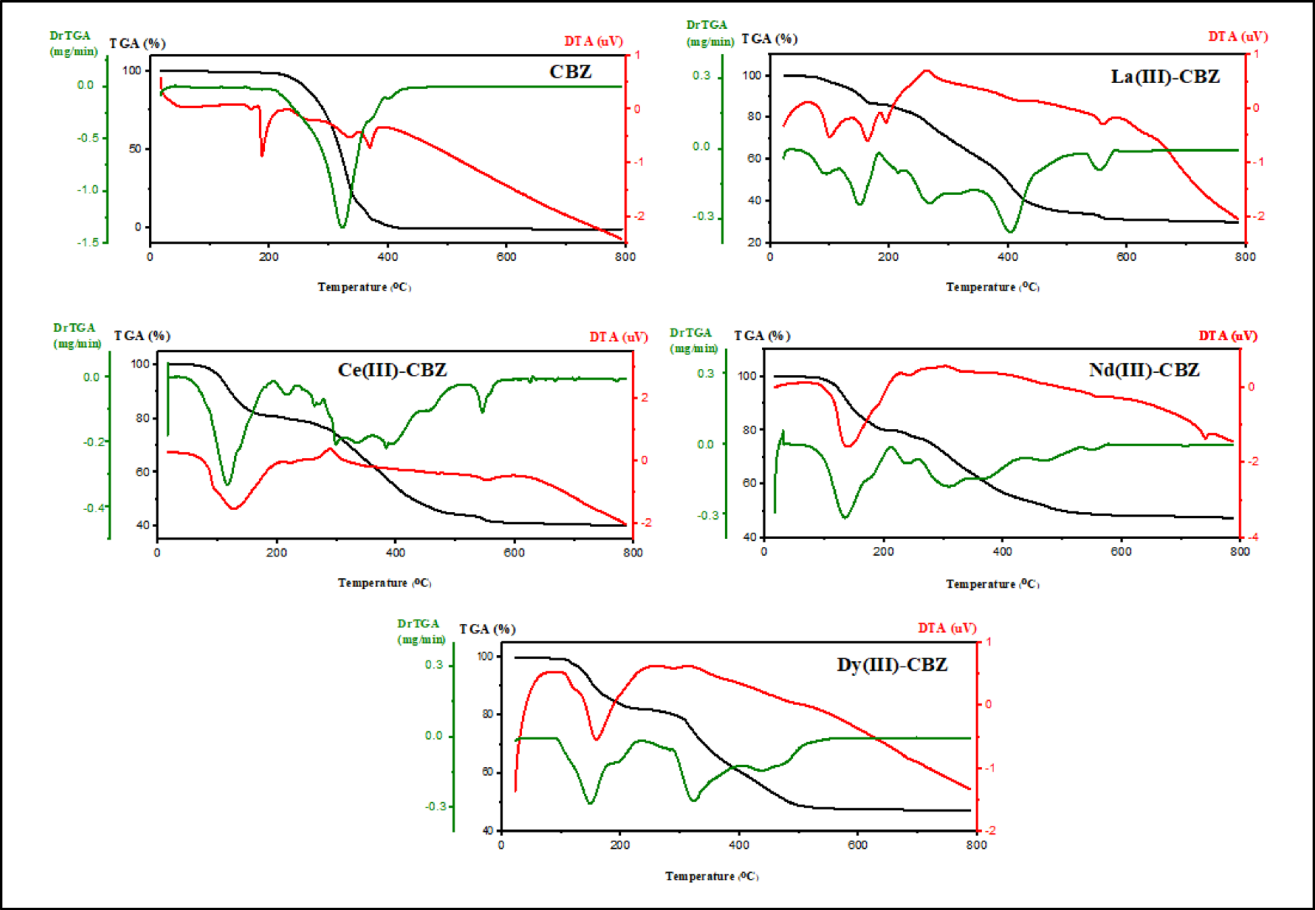
Thermal graphs for lanthanide complexes with CBZ ligands.

Thermogravimetry (TGA) is an analytical method that tracks how a substance’s weight changes over time or with temperature. Thermogravimetry requires a precise balance and a furnace with a linear temperature rise. The data can be displayed as a thermogravimetric (TGA) curve, or as a derivative thermogravimetric (DrTGA) curve, where the TGA curve’s first derivative is plotted versus time or temperature^49^. Using thermogravimetric (TGA, DrTGA and DTA) techniques (Fig. 7), the ligand’s and its complexes’ thermal properties were described throughout a temperature range of ambient temperature to 800 °C using a heating rate of 20 °C/min in an N_2_ environment. These techniques provide insight into the thermal stability of the newly synthesized complexes and determine whether any water molecules are situated within or beyond the complex’s inner coordination sphere. Table 5 lists the temperature ranges and the mass loss % for the ligand and its novel complexes. TGA curve of CBZ showed that it was completely decomposed in one step, this decomposition step occurred at the temperature range of 30–427 °C with weight loss 100% (Calc. 100%), which is related to the removal of C_15_H_12_N_2_O moiety. The DTA curve of the ligand (CBZ) shows three endothermic peaks at 186, 334, and 370 °C. TGA curve of the complex La(III)-CBZ showed two degradation steps in the temperature range 30–800 °C, the first step involves the loss of Cl_2_ gas and hydrated water molecule with mass loss 12.4% (Calc. 11.8%) at 30–162 °C, whereas the final second step occurred in the temperature range 162–570 °C with weight loss 55.7% (Calc. 55%) that corresponding to the removal of C_24_H_14_N_3_O, HCl and coordinated water molecule, leaving La + C_6_H_9_NO (33.2%) as a residue, these losses appear in the DTA data as four endothermic peaks at 98, 163, 195 and 559 and two exothermic peaks at 64 and 262 °C. TGA curve of the complex Ce(III)-CBZ showed two decomposition stages within the temperature range 30–800 °C, the first step was occurred at 30–210 °C with weight loss 19.6% (Calc. 20.4%) which corresponding to the removal of HCl, Cl_2_ gas and three water molecules of hydration, the second step was occurred at 210–566 °C with mass loss of 38.8% (Calc. 38.3%) which corresponding to removal of C_19_H_13_N_2_O and one coordinated water molecule, leaving Ce + C_11_H_10_N_2_O (41.2%) as a residue, theses mass losses were appeared in the DTA curve as two endothermic peaks at 121, 552 °C and one exothermic peak at 290 °C; on the other hand, the thermogram of the complex Nd(III)-CBZ showed that it was decomposed in two stages within the temperature range 20–800 °C. The first stage occurred at 20–210 °C with a weight loss of 20% (Calc. 20.8%) that related to the removal of HCl, Cl_2_ gas, two water molecules of hydration, and one coordinated water molecule. The second stage occurred from 210 to 555 with mass loss of 31.3% (Calc. 30.2%) which attributed to the elimination of C_15_H_11_N_2_O moiety, the Nd + C_15_H_12_N_2_O was left as a residue with a percentage (49%), the DTA curve showed three endothermic peaks at 136, 240 and 738 °C; finally, TGA curve of the complex Dy(III)-CBZ showed two decomposition steps within the temperature range 40–800 °C. The first step occurred at 40–240 °C with weight loss 17.9% (Calc. 18.5%), corresponding to the removal of HCl, Cl_2_ gas, hydrated and coordinated water molecules, the second step of decomposition occurred at 240–546 °C with mass loss 34.2% (Calc. 33.3%) related to the removal of C_17_H_11_N_2_O, leaving Dy + C_13_H_12_N_2_O (48.2%) as a residue. Based on the data provided by the DTA, it is evident that these mass losses coincide with an endothermic peak at 155 °C and 288 °C.

**Table 5:** Data from thermal analysis of lanthanide complexes with CBZ ligand.

Complex	Degradation steps	Tem. Rang (^o^C)	Weight loss (%)		Assignment
			Calc.	Found	
C_15_H_12_N_2_O	First step Total loss Residue	150–800	100 100 0.00	100 100 0.00	Completely decomposed (C_15_H_12_N_2_O)
C_30_H_28_LaCl_3_N_4_O_4_	First step Second step residue	30–162 162–570 > 570	11.8 55 33.2	12.4 55.7 32	H_2_O_hyd_. + Cl_2_ H_2_O_coord_. +HCl + C_24_H_14_N_3_O La + C_6_H_9_NO
C_30_H_32_CeCl_3_N_4_O_6_	First step Second step Residue	30–210 210–566 > 566	20.4 38.3 41.2	19.6 38.8 41.6	3H_2_O_hyd_. + Cl_2_ + HCl H_2_O_coord_. + C_19_H_13_N_2_O Ce + C_11_H_10_N_2_O
C_30_H_30_NdCl_3_N_4_O_5_	First step Second step residue	20–210 210–555 > 555	20.8 30.2 49	20 31.3 48.7	2H_2_O_hyd_. + Cl_2_ + HCl + H_2_O_coord_. C_15_H_11_N_2_O Nd + C_15_H_12_N_2_O
C_30_H_28_DyCl_3_N_4_O_4_	First step Second step Residue	40–240 240–546 > 546	18.5 33.3 48.2	17.9 34.2 48	H_2_O_hyd_. + Cl_2_ + HCl + H_2_O_coord_. C_17_H_11_N_2_O Dy + C_13_H_12_N_2_O

### Theoretical studies

#### Molecular DFT calculation of the (CBZ)

**Fig. 8:**
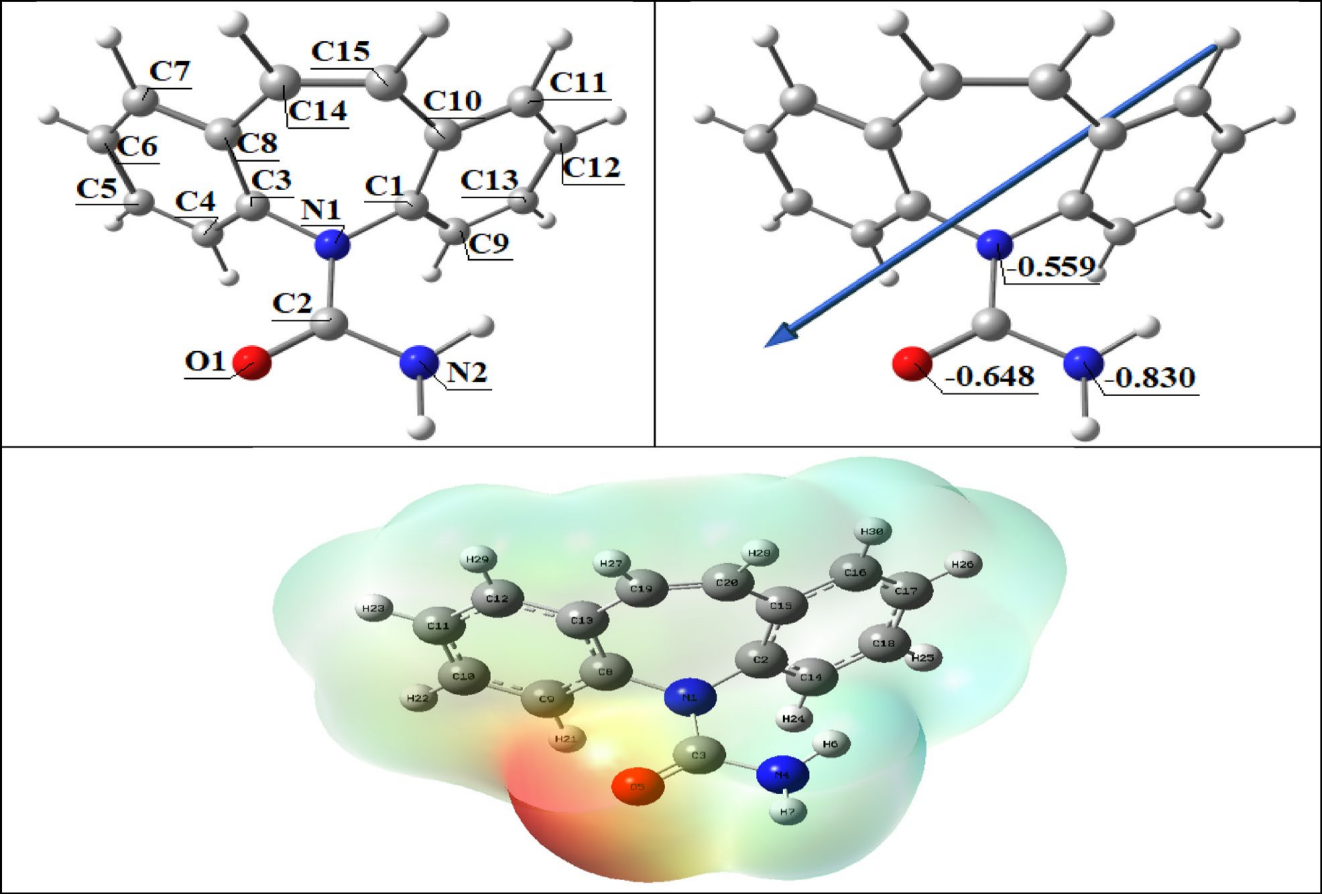
The optimized ligand structure, the dipole moment vector, the natural charges on atoms, and the molecular electrostatic potential (MEP) surface by density function B3LYP/6–311 + + g(d, p).

Figure 8 shows the configurations with the lowest energy as the ligand’s ideal structures. According to Natural Bond Orbital Analysis’s (NBO) natural charges, the more negatively charged active sites are O1 (−0.648), N1 (−0.559), and N2 (−0.830). Metal ions may coordinate with atoms O1 and N2.

#### Molecular DFT complex calculation

**Fig. 9:**
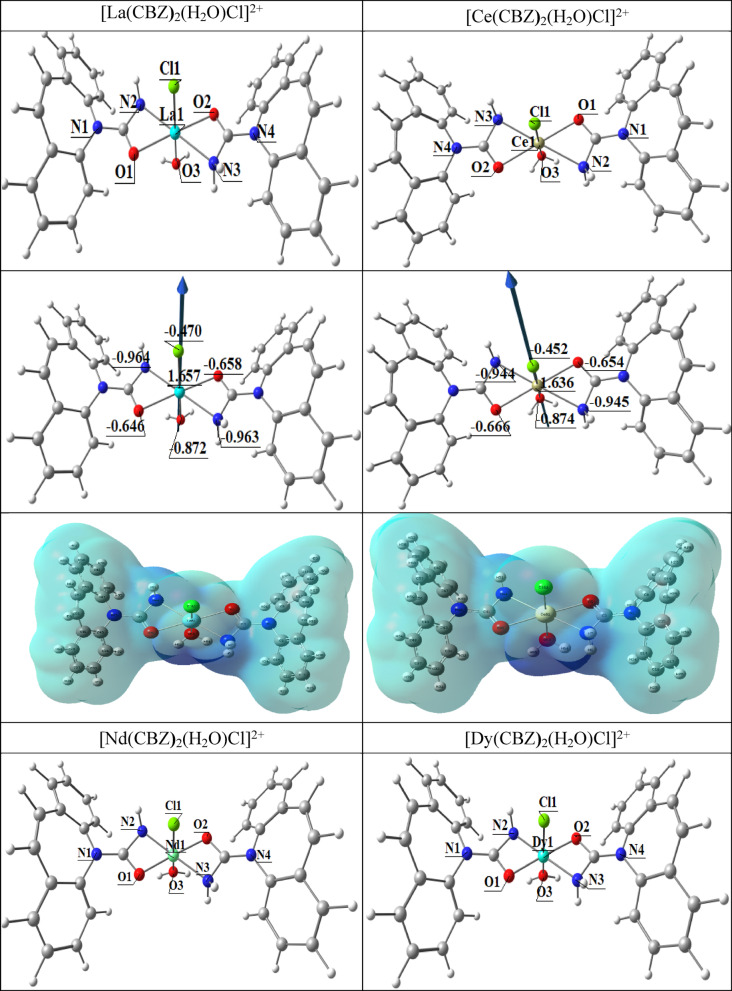

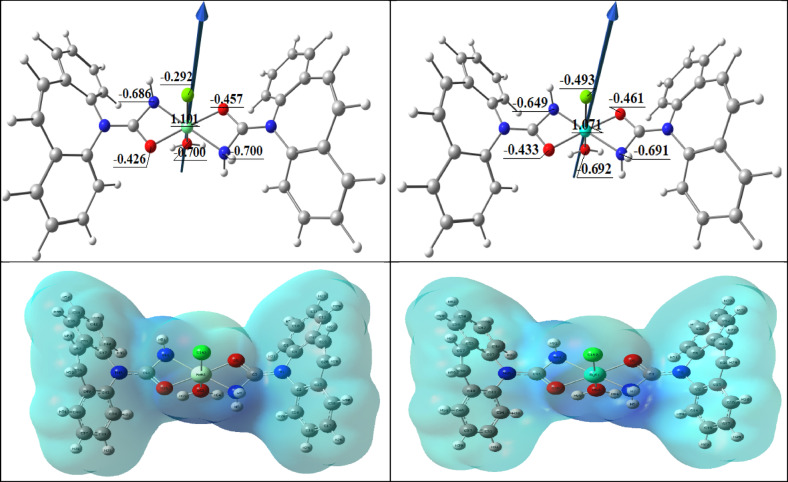
The dipole moment vector, the optimal structures, and the natural charges on coordinated atoms of [La(CBZ**)**_2_(H_2_O)Cl]^2+^ (I), [Ce(CBZ**)**_2_(H_2_O)Cl]^2+^ (II), [Nd(CBZ**)**_2_(H_2_O)Cl]^2+^ (III), and [Dy(CBZ**)**_2_(H_2_O)Cl]^2+^ (IV) complexes.

Figure 9 displays the complexes [La(CBZ)_2_(H_2_O)Cl]^2+^ (I), [Ce(CBZ)_2_(H_2_O)Cl]^2+^ (II), [Nd(CBZ)_2_(H_2_O)Cl]^2+^ (III), and [Dy(CBZ)_2_(H_2_O)Cl]^2+^ (IV) optimal lowest energy forms; respectively. For every complex, the metal atoms create six-coordinated deformed octahedral structures. The atoms O1, N2, O2, and N3 are deviated by −3.906°, −5.634, −4.782, and − 4.225° in the complexes [La(CBZ)_2_(H_2_O)Cl], [Ce(CBZ)_2_(H_2_O)Cl], [Nd(CBZ)_2_(H_2_O)Cl], and [Dy(CBZ)_2_(H_2_O)Cl]; respectively, Table 6. Also Fig. 9. displays the complexes’ [La(CBZ)_2_(H_2_O)Cl]^2+^ (I), [Ce(CBZ)_2_(H_2_O)Cl]^2+^ (II), [Nd(CBZ)_2_(H_2_O)Cl]^2+^ (III), and [Dy(CBZ)_2_(H_2_O)Cl]^2+^ (IV) optimal structures, the natural charges on their coordinated atoms and the dipole moment vector. The atoms’ natural charges from the NBO analysis were coordinated for [La(CBZ)_2_(H_2_O)Cl]^2+^ are {La(+ 1.657), N2(−0.964), N3(−0.963), O1(−0.646), O2(−0.658), O3(−0.872) and Cl(−0.470)}, for [Ce(CBZ)_2_(H_2_O)Cl]^2+^ are {Ce(+ 1.636), N2(−0.945), N3(−0.944), O1(−0.654), O2(−0.666), O3(−0.847) and Cl(−0.452), for [Nd(CBZ)_2_(H_2_O)Cl]^2+^ are {Nd(+ 1.101), N2(−0.686), N3(−0.700), O1(−0.426), O2(−0.457), O3(−0.700) and Cl(−0.292) and for [Dy(CBZ)_2_(H_2_O)Cl]^2+^ are {Dy(+ 1.071), N2(−0.649), N3(−0.691), O1(−0.433), O2(−0.461), O3(−0.692) and Cl(−0.493)}.

**Fig. 10:**
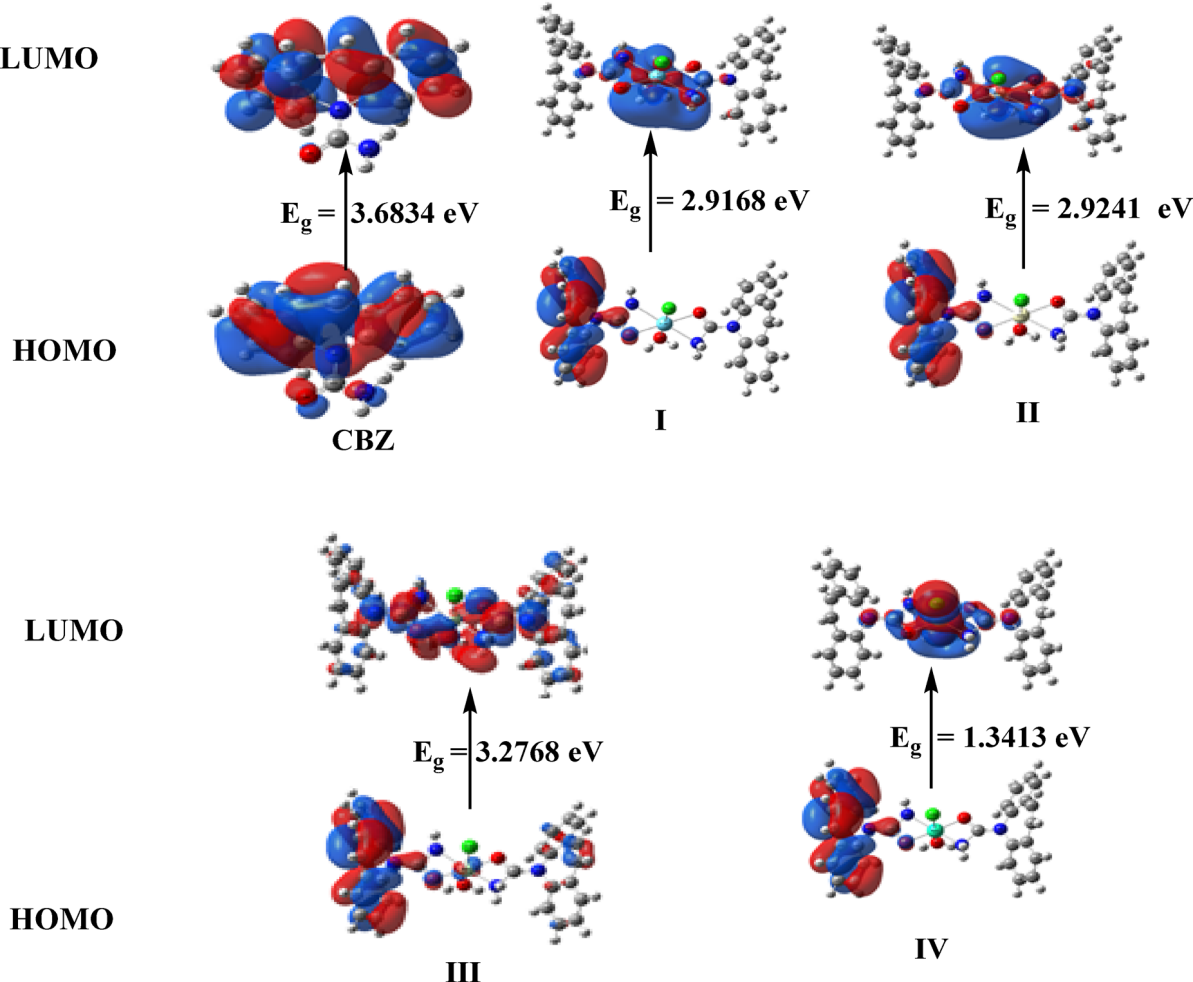
Ligand’s HOMO and LUMO charge density maps, [La(CBZ**)**_2_(H_2_O)Cl]^2+^ (I), [Ce(CBZ**)**_2_(H_2_O)Cl]^2+^ (II), [Nd(CBZ**)**_2_(H_2_O)Cl]^2+^ (III), and [Dy(CBZ**)**_2_(H_2_O)Cl]^2+^ (IV).

Table 7 displays the calculated total energy, the dipole moment, and the energies of the ligands and complexes’ highest occupied molecular orbital (HOMO) and lowest unoccupied molecular orbital (LUMO). The energy gap (Eg) equals E_LUMO_ – E_HOMO_ is less in the case of complexes than that of the ligand because of chelation of the ligand to metal ions, and the bigger negative values of the complexes’ total energy relative to the free ligand indicate that the complexes are more stable than the free ligand, Table 7. The lower quantity of Eg in complexes when compared to the ligand clarifies the charge transfer interactions during compound formation. (Fig. 10).

#### Reactivity studies

Numerous reactivity descriptors derived from HOMO and LUMO energies have been proposed to help understand various aspects of reactivity in chemical processes. Electron affinity (A), hardness (η), ionization potential (I), chemical potential (µ), softness (S), electronegativity (χ), and electrophilicity index (ω) are some of these. Table 7.

**Table 6:** Significant optimal bond angles (°) and bond lengths (Å) of the complexes [La(CBZ**)**_2_(H_2_O)Cl]^2+^ (I), [Ce(CBZ**)**_2_(H_2_O)Cl]^2+^ (II), [Nd(CBZ**)**_2_(H_2_O)Cl]^2+^ (III), and [Dy(CBZ**)**_2_(H_2_O)Cl]^2+^ (IV).

Bond lengths	I	II	III	IV
M-O1	2.349	2.353	1.914	1.936
M-O2	2.348	2.352	1.974	1.964
M-N2	2.421	2.494	2.026	1.937
M-N3	2.416	2.485	2.094	2.035
M-Cl	2.723	2.674	2.605	2.470
M-O3	2.517	2.552	1.887	1.847
**Angles**	**I**	**II**	**III**	**IV**
O1-M-N2	57.96	56.83	63.08	63.13
O2-M-N3	58.03	56.94	61.69	61.59
O1-M-N3	121.3	122.5	116.1	116.1
O2-M-N2	122.6	123.8	119.1	119.2
O3-M-O1	88.85	91.34	92.19	91.80
O3-M-O2	88.72	88.31	87.80	87.58
O3-M-N2	91.04	91.34	89.06	87.93
O3-M-N3	90.34	90.22	95.64	95.63
Cl-M-O1	91.30	92.02	88.09	88.45
Cl-M-O2	91.13	91.87	91.91	92.17
Cl-M-N2	89.23	89.11	91.21	92.14
Cl-M-N3	89.38	89.34	84.09	84.31
Cl-M-O3	179.7	179.5	179.7	179.7
O1-M-O2	177.5	176.1	177.8	177.6
N2-M-N3	178.4	178.3	175.3	176.4
O1-N2-O2-N3	−3.906*	−5.634*	−4.782*	−4.225*

*dihedral angle.

**Table 7:** Calculated ligand and complex energies and characteristics [La(CBZ**)**_2_(H_2_O)Cl]^2+^ (I), [Ce(CBZ**)**_2_(H_2_O)Cl]^2+^ (II), [Nd(CBZ**)**_2_(H_2_O)Cl]^2+^ (III), and [Dy(CBZ**)**_2_(H_2_O)Cl]^2+^ (IV).

Property	CBZ	I	II	III	IV
E(a.u.)	−763.769	−2499.059	−2538.569	−2624.790	−2765.206
HOMO (eV)	−6.2004	−10.9067	−10.9048	−11.0846	−11.0204
LUMO (eV)	−1.7761	−7.8195	−7.6372	−9.1480	−9.2862
E_g_(eV)	4.4243	3.0872	3.2676	1.9366	1.7342
Dipole moment (Debye)	3.8192	9.3692	9.1277	8.5207	7.0687
I=-E_HOMO_	6.2004	10.9067	10.9048	11.0846	11.0204
A=-E_LUMO_	1.7761	7.8195	7.6372	9.148	9.2862
χ=(I +A)/2	3.9882	9.3631	9.2710	10.1163	10.1533
η=(I -A)/2	2.2121	1.5436	1.6338	0.9683	0.8671
S=1/2η	0.2260	0.3239	0.3060	0.5164	0.5766
μ=-χ	−3.9882	−9.3631	−9.2710	−10.1163	−10.1533
ω=μ^2^/2η	3.5951	28.3971	26.3041	52.8450	59.4450

### Biological processes

#### Antimicrobial efficacy

**Fig. 11:**
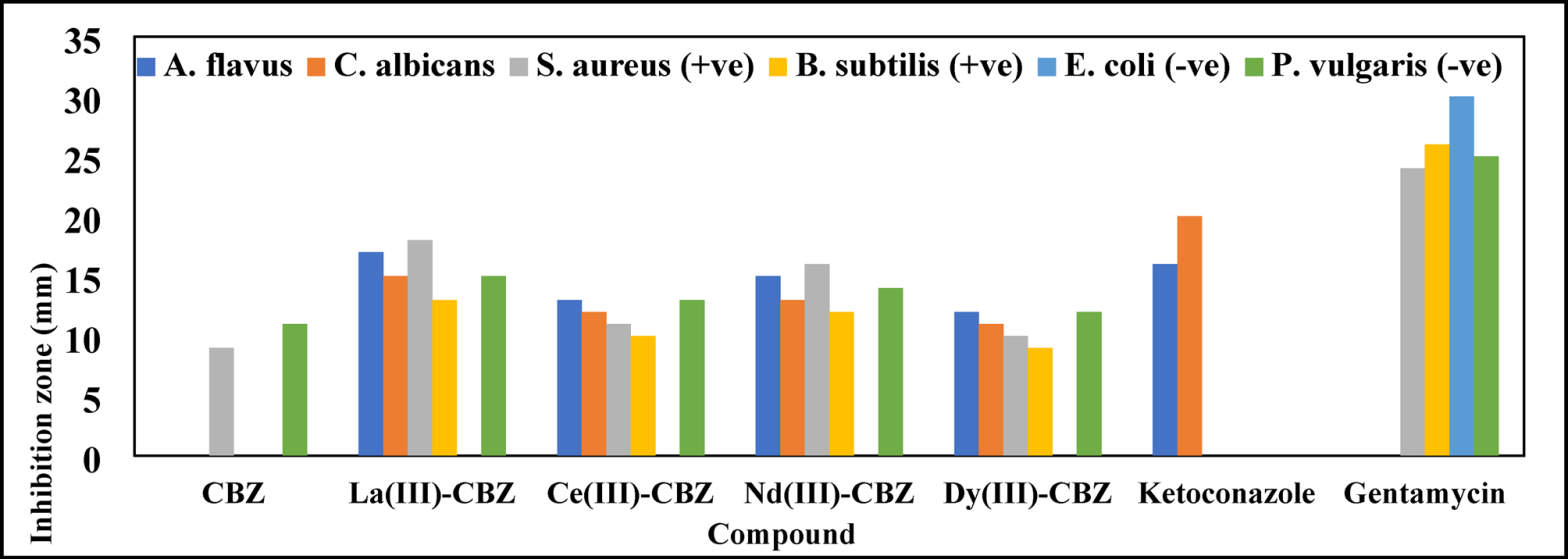
CBZ ligand and lanthanide complexes’ antimicrobial properties.

The antibacterial and antifungal properties of the ligand and its lanthanide complexes against a range of pathogenic microbes, including two Gram-positive bacteria (*Bacillus subtilis* and *Staphylococcus aureus*), two Gram-negative bacteria (*Proteus vulgaris* and *Escherichia coli*), and anti-fungal efficacy (*Candida albicans* and *Aspergillus flavus*), were evaluated using the diffusion agar technique^50^ Fig. 11. The antibacterial efficiency and mean zone of inhibition in millimeters produced by ligand and its complexes on a range of harmful pathogens are shown in Table S1. Based on the acquired results; we notice that, the ligand (CBZ) and its synthesized complexes have no antibacterial properties with *Escherichia coli* (Gram-negative bacteria), on the other hand, all complexes have antibacterial activity against *Bacillus subtilis* (Grame positive bacteria) with inhibition zone (13, 10, 12, and 9 mm for the complexes of La(III), Ce(III), Nd(III), and Dy(III), respectively) but ligand (CBZ) showed no antibacterial activity against *Bacillus subtilis*. There was no antifungal effect of the ligand towards *Aspergillus flavus* and *Candida albicans*. The complexes have been arranged according to their antibacterial and antifungal activity as follows: La-CBZ > Nd-CBZ > Ce-CBZ > Dy-CBZ > CBZ. Among all complexes, the La (III) complex demonstrated high antibacterial and antifungal activity, with its inhibitory zone significantly larger than that of ketoconazole against *Aspergillus flavus*. In contrast, the Nd (III) complex exhibited an inhibition zone nearly equal to that of ketoconazole, measuring 15 mm compared to 16 mm for ketoconazole.

#### Analyzing the anticancer activity in vitro

**Fig. 12:**
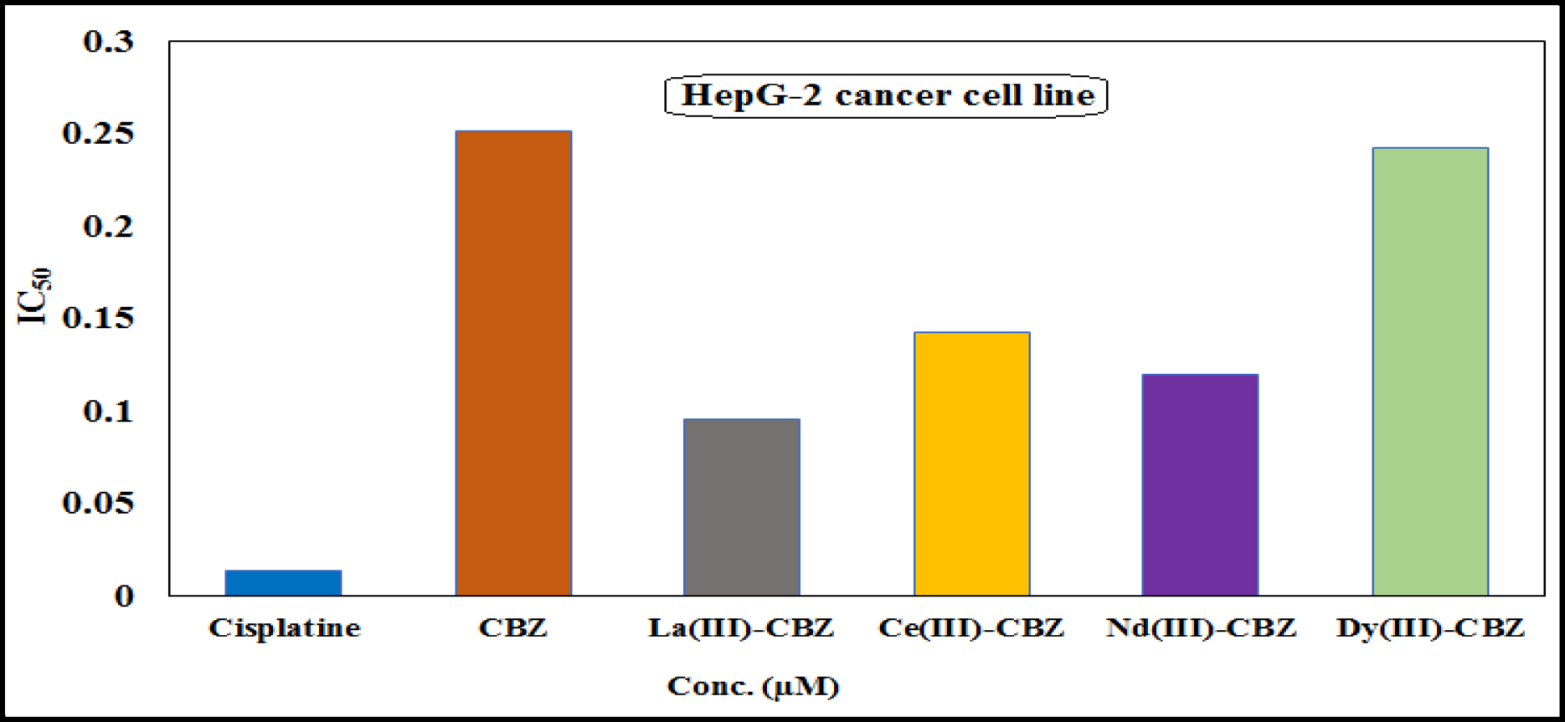
CBZ ligand and its lanthanide complexes’ IC_50_ values towards the HepG-2 cell line.

**Fig. 13:**
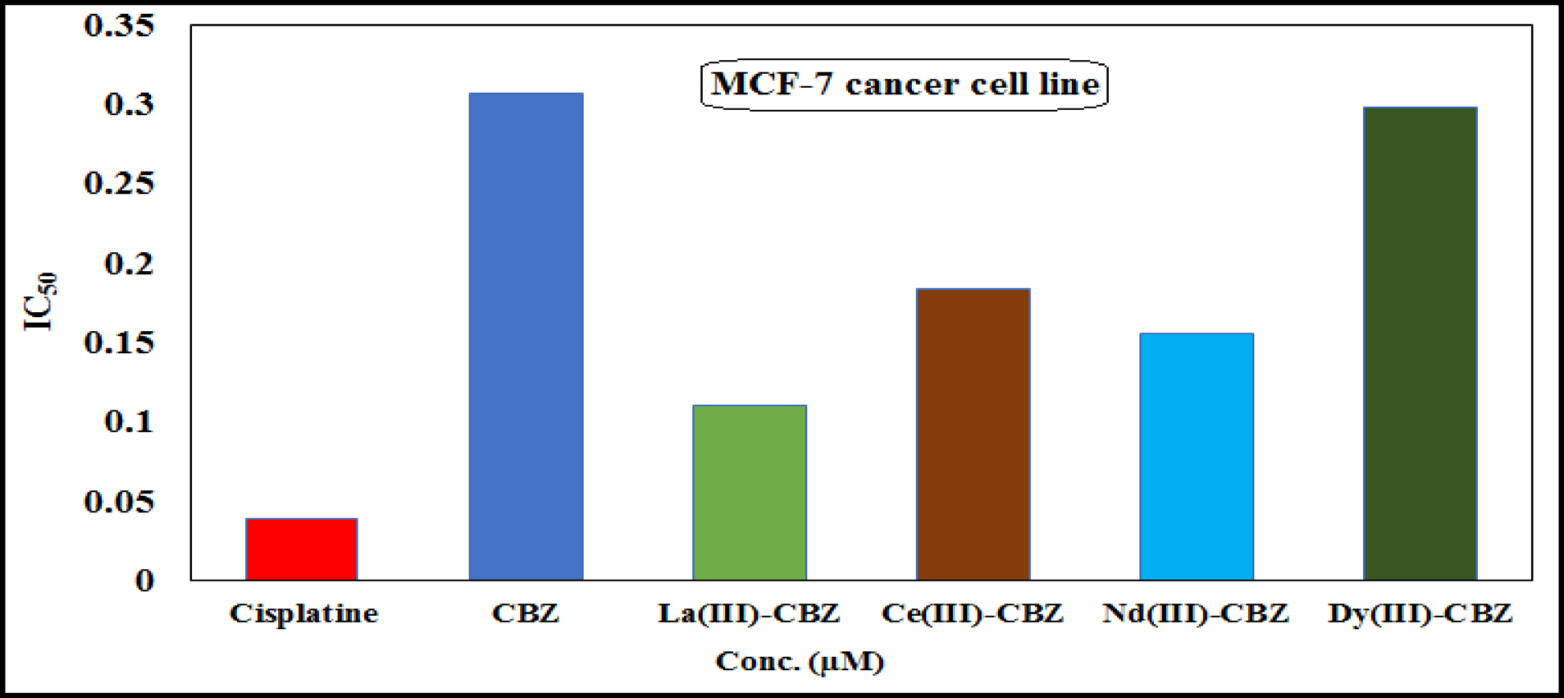
CBZ ligand and its lanthanide complexes’ IC_50_ values towards the MCF-7 cell line.

Using the MTT assay, the anticancer efficacy of CBZ ligand and its lanthanide complexes [La(III), Ce(III), Nd(III), and Dy(III)] was found in vitro on two distinct human cancer cell lines (Hep-G2 and MCF-7)^51^. The data’s cytotoxicity of CBZ and the newly synthesized complexes versus liver and breast cancer cell lines at dosages of 3.9, 7.8, 15.6, 31.25, 62.5, 125, 250, and 500 µg/ml is mentioned in Table S2. The IC_50_ values of CBZ and its complexes against Hep-G2 and MCF-7, compared with cisplatin as a reference, are shown in Figs. 12 and 13. From the obtained results; it is showed that the La(III) complex (IC_50_ = 0.095 and 0.110 µM) demonstrated a high degree of activity against Hep-G2 and MCF-7 cancer cell lines, the IC_50_ values showed that all synthesized complexes have high ability rather than free ligand to inhibit the growth of all breast and human liver cancer cell lines compared with cisplatin as a standard drug (IC_50_ = 0.013 and 0.039 µM). The cytotoxic activity of CBZ and the new complexes can be ordered depending on their IC_50_ values as follows: La-CBZ > Nd-CBZ > Ce-CBZ > Dy-CBZ > CBZ. The complexes that have been prepared are more effective towards the human cancer cell lines, which are considered a good inhibitor, dependent on their concentration, which may have a significant impact on cancer treatment.

#### Molecular Docking of complexes

Using molecular docking, ligand (CBZ), La(III), Ce(III), Nd(III), and Dy(III) complexes with Bacillus subtilis receptor proteins (PDB ID: 6A4M) and the receptor of methionine adenosyltransferases in hepatic carcinoma (PDB ID: 5A19) and human breast cancer MCF-7 (PDB ID: 4zvm) provide important insights into their possible antibacterial and anticancer mechanisms. These simulations demonstrate how the metal complexes affect the activity of important bacterial enzymes or membrane proteins by interacting with their active sites. Through hydrogen bonding, π-π stacking, and coordination interactions with amino acid residues, the ligand (CBZ) enhances the binding affinity. Higher binding energies are frequently seen in metal complexes involving Dy(III) and Ce(III), indicating more persistent interactions with the bacterial and cancer receptors. These results suggest that the mechanism of action probably involves disruption of essential biochemical pathways in B. subtilis (PDB ID: 6A4M) and the methionine adenosyltransferase receptor in hepatic carcinoma (PDB ID: 5A19) and human breast cancer MCF-7 (PDB ID: 4zvm), thereby impairing its growth and survival. The concept that these complexes can be useful for antibacterial applications in addition to their capacity to combat cancer is supported by the docking data.

The ligand’s binding free energy and complexes with the Bacillus subtilis receptor (PDB ID: 6A4M) are found to be − 7.2, −18.1, −15.0, −17.4 and − 13.6 kcal/mol for CBZ, [La(CBZ**)**_2_(H_2_O)Cl]^2+^, [Ce(CBZ**)**_2_(H_2_O)Cl]^2+^, [Nd(CBZ**)**_2_(H_2_O)Cl]^2+^, and [Dy(CBZ**)**_2_(H_2_O)Cl]^2+^; respectively, Table 8. The interaction is stronger when the binding energy is more negative.

CBZ˂[Dy(CBZ**)**_2_(H_2_O)Cl]^2+^˂[Ce(CBZ**)**_2_(H_2_O)Cl]^2+^˂[Nd(CBZ**)**_2_(H_2_O)Cl]^2+^˂[La(CBZ**)**_2_(H_2_O)Cl]^2+^.

**Table 8:** Docking interaction data computations of CBZ, [La(CBZ**)**_2_(H_2_O)Cl]^2+^, [Ce(CBZ**)**_2_(H_2_O)Cl]^2+^, [Nd(CBZ**)**_2_(H_2_O)Cl]^2+^, and [Dy(CBZ**)**_2_(H_2_O)Cl]^2+^ with the active sites of Bacillus subtilis (PDB ID: 6A4M).

	Receptor	Interaction	Distance(Å)*	E (kcal/mol)	S score	RMSD
CBZ						
N 4	OD1 ASP 115	H-donor	2.89 (2.01)	−3.6		
N 4	O ILE 116	H-donor	3.35 (2.46)	−1.0		
O 5	N PHE 118	H-acceptor	3.33 (2.46)	−1.1	−5.477	1.015
7-ring	CD ARG 36	pi-H	4.09	−1.0		
6-ring	CD ARG 36	pi-H	4.15	−0.5		
[La(CBZ**)**_2_(H_2_O)Cl]^2+^						
N 4	OE2 GLU 306	Ionic	2.96	−11.7	−9.131	1.122
N 43	OXT GLU 309	Ionic	3.17	−6.4		
[Ce(CBZ**)**_2_(H_2_O)Cl]^2+^						
N 1	OE1 GLU 114	Ionic	3.61	−1.5		
N 1	OE2 GLU 114	Ionic	3.63	−1.4		
N 4	OE1 GLU 114	Ionic	2.91	−5.1	5.861	1.539
N 4	OE2 GLU 114	Ionic	3.57	−1.6		
CE 60	NH2 ARG 143	Ionic	2.87	−5.4		
[Nd(CBZ**)**_2_(H_2_O)Cl]^2+^						
N 4	OD1 ASP 37	H-donor	3.24 (2.50)	−2.4		
O 61	N ASP 37	H-acceptor	2.90 (1.90)	−5.2		
N 4	OD1 ASP 37	Ionic	3.24	−4.0	−6.907	1.217
N 4	OD2 ASP 37	Ionic	3.08	−5.0		
6-ring	N ALA 34	pi-H	3.90	−0.8		
[Dy(CBZ**)**_2_(H_2_O)Cl]^2+^						
CL 60	NH1 ARG 229	H-acceptor	3.05 (2.30)	−4.5		
N 4	OE2 GLU 119	Ionic	3.19	−2.3		
N 43	OE2 GLU 226	Ionic	3.46	−1.1	−5.438	1.345
DY 59	NH1 ARG 229	Ionic	3.85	−0.8		
DY 59	NH2 ARG 229	Ionic	2.89	−4.3		
6-ring	N ALA 120	pi-H	4.11	−0.6		

* H-bond lengths are enclosed in brackets.

**Fig. 14:**
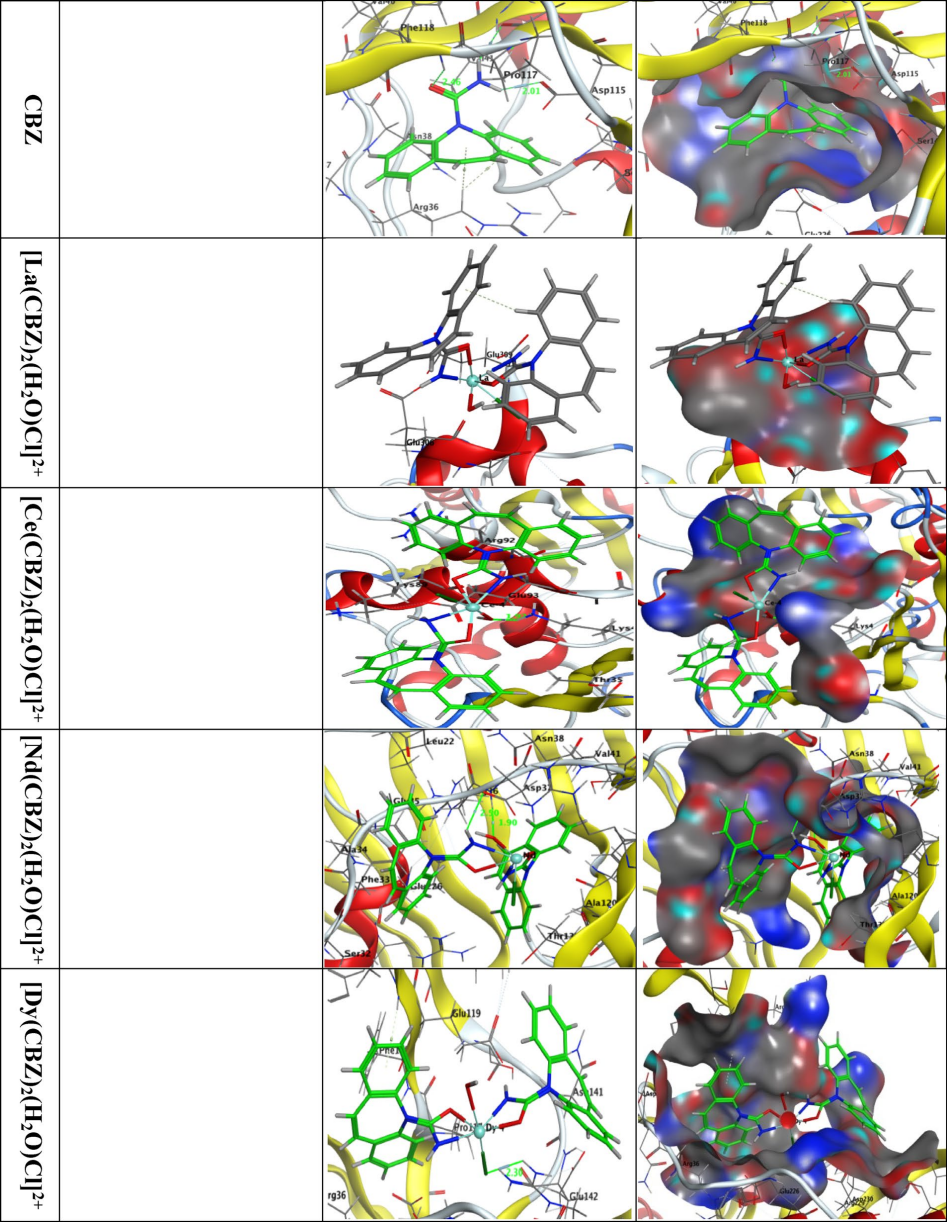
displays the 2D and 3D plots showing how CBZ, [La(CBZ)_2_(H_2_O)Cl]^2+^, [Ce(CBZ)_2_(H_2_O)Cl]^2+^, [Nd(CBZ)_2_(H_2_O)Cl]^2+^, and [Dy(CBZ)_2_(H_2_O)Cl]^2+^ interact with the active site of the Bacillus subtilis receptor (PDB ID: 6A4M).

Figure 14. Plots in two and three dimensions showing how CBZ, [La(CBZ)_2_(H_2_O)Cl]^2+^, [Ce(CBZ)_2_(H_2_O)Cl]^2+^, [Nd(CBZ)_2_(H_2_O)Cl]^2+^, and [Dy(CBZ)_2_(H_2_O)Cl]^2+^ interact with the Bacillus subtilis active site (PDB ID: 6A4M). Dotted curves illustrate hydrophobic interactions with amino acid residues.

- The free energy of the CBZ ligand’s binding and complexing with the methionine adenosyltransferase receptor in human breast cancer MCF-7 (PDB ID: 4zvm) and hepatic carcinoma (PDB ID: 5A19) is shown to be (−2.8, −17.9, −13.9, −14.6 and − 13.7) and (−2.4, −15.8, −12.7, −13.7 and − 11.3) kcal/mol for the 1for CBZ, [La(CBZ**)**_2_(H_2_O)Cl]^2+^, [Ce(CBZ**)**_2_(H_2_O)Cl]^2+^, [Nd(CBZ**)**_2_(H_2_O)Cl]^2+^, and [Dy(CBZ**)**_2_(H_2_O)Cl]^2+^; respectively, (Table 9 and S3). Greater negative binding energies result in stronger interactions.

CBZ˂[Dy(CBZ**)**_2_(H_2_O)Cl]^2+^˂[Ce(CBZ**)**_2_(H_2_O)Cl]^2+^˂[Nd(CBZ**)**_2_(H_2_O)Cl]^2+^˂[La(CBZ**)**_2_(H_2_O)Cl]^2+^.

**Table 9:** CBZ, [La(CBZ**)**_2_(H_2_O)Cl]^2+^, [Ce(CBZ**)**_2_(H_2_O)Cl]^2+^, [Nd(CBZ**)**_2_(H_2_O)Cl]^2+^, and [Dy(CBZ**)**_2_(H_2_O)Cl]^2+^ Docking interaction data calculations with the active sites of the liver cancer protein receptor (PDB ID: 5A19).

	Receptor	Interaction	Distance(Å)*	E (kcal/mol)	S score	RMSD
CBZ						
N 4	O GLY 381	H-donor	2.99(1.98)	−1.2		
7-ring	NZ LYS 303	pi- cation	4.91	−0.8	−4.326	1.234
6-ring	NZ LYS 303	pi-cation	3.66	−0.8		
[La(CBZ**)**_2_(H_2_O)Cl]^2+^						
LA 59	OXT TYR 395	H-donor	2.47	−8.6		
CL 60	NZ LYS 351	H-acceptor	3.36 (2.39)	−2.5		
N 4	OXT TYR 395	Ionic	3.20	−2.3	−7.233	1.541
6-ring	NZ LYS 340	pi-cation	4.05	−0.8		
6-ring	NZ LYS 340	pi-cation	3.23	−2.6		
6-ring	NZ LYS 351	pi-cation	4.44	−1.1		
[Ce(CBZ**)**_2_(H_2_O)Cl]^2+^						
CL 61	NZ LYS 307	H-acceptor	2.91 (1.99)	−7.9		
CL 61	SG CYS 149	H-donor	4.31	−0.9	−6.270	1.157
CE 60	NZ LYS 307	Ionic	2.92	−5.1		
[Nd(CBZ**)**_2_(H_2_O)Cl]^2+^						
N 43	OE1 GLU 85	H-donor	3.60 (2.58)	−2.1	−6.376	1.371
N 43	OE1 GLU 85	Ionic	3.60	−12.5		
[Dy(CBZ**)**_2_(H_2_O)Cl]^2+^						
O 61	NH1 ARG 169	H-acceptor	2.97 (1.99)	−8.3		
O 61	CD ARG 169	H-acceptor	3.47 (2.39)	−0.6		
N 4	OE2 GLU 27	Ionic	3.98	−0.6	−5.179	1.098
DY 59	NE ARG 169	Ionic	3.79	−1.0		
DY 59	NH1 ARG 169	Ionic	3.38	−2.4		
6-ring	CE2 TYR 377	pi-H	4.32	−0.8		

* H-bond lengths are enclosed in brackets.

Fig.s 15 and S3 display The two-dimensional and three-dimensional representations of CBZ interaction, [La(CBZ**)**_2_(H_2_O)Cl]^2+^, [Ce(CBZ**)**_2_(H_2_O)Cl]^2+^, [Nd(CBZ**)**_2_(H_2_O)Cl]^2+^, and [Dy(CBZ**)**_2_(H_2_O)Cl]^2+^ with the active site of the receptor of breast cancer MCF-7 (PDB ID: 4zvm) and hepatocellular carcinoma protein (PDB ID: 5A19).

**Fig. 15:**
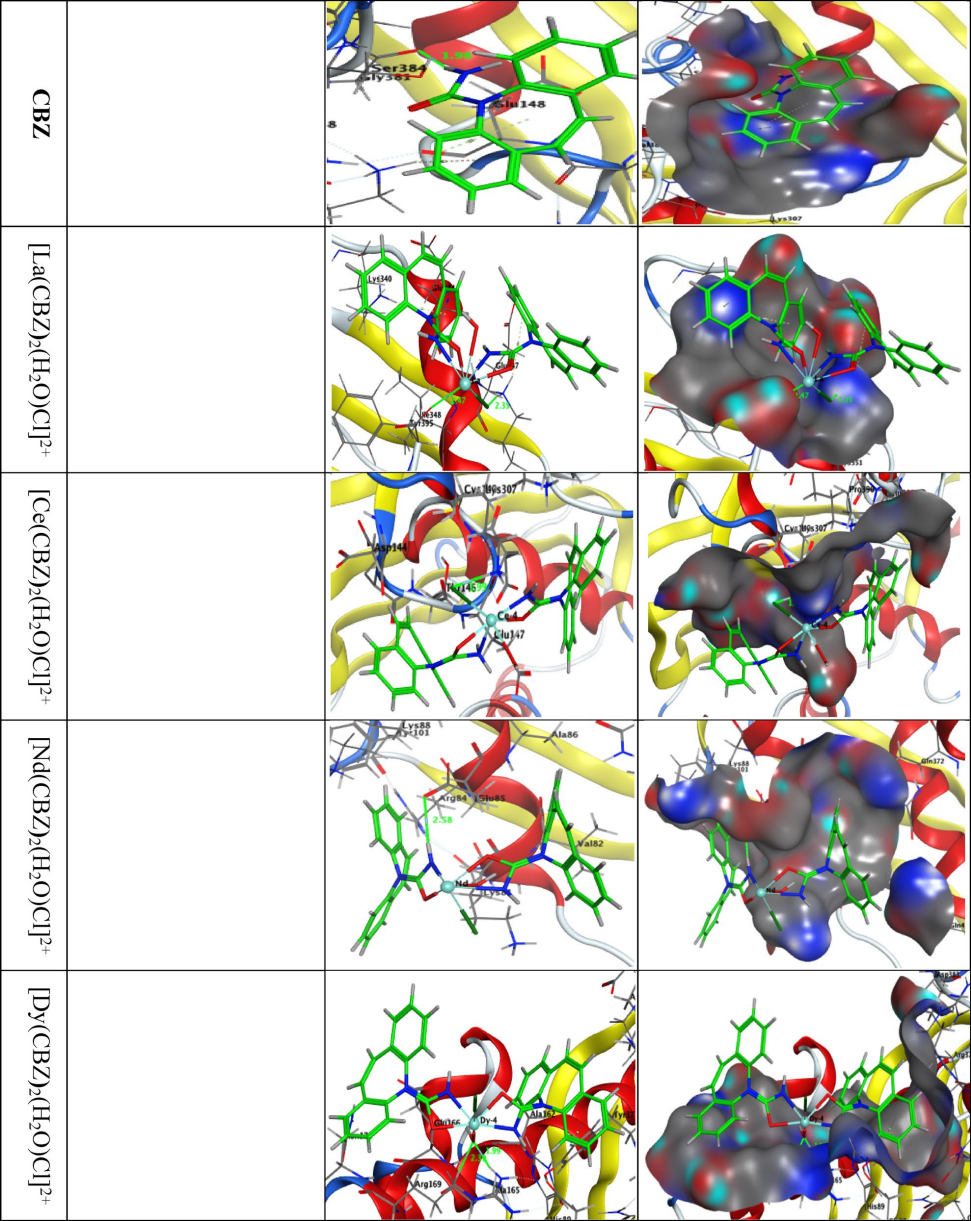
Plots in two and three dimensions that illustrate the interactions between CBZ, [La(CBZ)_2_(H_2_O)Cl]^2+^, [Ce(CBZ)_2_(H_2_O)Cl]^2+^, [Nd(CBZ)_2_(H_2_O)Cl]^2+^, and [Dy(CBZ)_2_(H_2_O)Cl]^2+^ with the liver cancer protein receptor’s active site (PDB ID: 5A19). Hydrophobic interactions with amino acid residues are depicted by dotted curves.

## Conclusions

Different lanthanide complexes, specifically [La(CBZ)_2_(H_2_O)Cl]Cl_2_∙H_2_O, [Ce(CBZ)_2_(H_2_O)Cl]Cl_2_∙3H_2_O, [Nd(CBZ)_2_(H_2_O)Cl]Cl_2_∙2H_2_O, and [Dy(CBZ)_2_(H_2_O)Cl]Cl_2_∙H_2_O, have been synthesized in a 1:2 molar ratio (Ln^3+^: CBZ) and characterized using various techniques. These octahedral geometry complexes have the carbamazepine ligand coordinating as a bidentate ligand through the nitrogen and oxygen of the amide group. Molar conductivity indicated their electrolytic behavior. Thermogravimetric analysis (TGA) validated the molecular formulas and thermal stability. DFT data confirmed that the novel stable complexes have good electronic characteristics for use in coordination chemistry’s application. These complexes have demonstrated significant activity regarding their antibacterial and antifungal properties, which La (III) complex in particular exhibiting the highest antibacterial and antifungal activity. The complexes studied have demonstrated a high potential for cytotoxic effect against a variety of cancer cell lines, including MCF-7 and HepG-2. It has been demonstrated that the La (III) complex revealed the highest activity towards the MCF-7 and HepG-2 cancer cell lines in contrast with cisplatin as a standard drug and the activity arrange as follow: La-CBZ > Nd-CBZ > Ce-CBZ > Dy-CBZ > CBZ. This suggests potential use as antimicrobial agents and alternative metal-based anticancer drugs, also indicating possible applications in bioimaging and drug delivery due to their unique electronic and coordination properties.

## Supplementary Information

Below is the link to the electronic supplementary material.

Supplementary Material 1

## Data Availability

The data presented in this study are available on request from the corresponding authors at norasaad@sci.nvu.edu.eg; Ehababdalla99@sci.nvu.edu.eg.
