# Supplementary material for: Lanthanide–carbamazepine complexes: synthesis, spectroscopic characterization, DFT Insights, molecular docking, and biological evaluation
Source: Sci Rep. 2026 Feb 11;16:6340. doi: 10.1038/s41598-026-35893-9 (PMC12905177; doi:10.1038/s41598-026-35893-9)
Supplement: Supplementary file 1 — Supplementary Material 1 [file 41598_2026_35893_MOESM1_ESM.docx]

**Lanthanide–Carbamazepine Complexes: Synthesis, Spectroscopic Characterization, DFT Insights, Molecular Docking, and Biological Evaluation**

**Nora S. Mohamed** ^1,*^**, Mahmoud M.A. Mohamed** ^1^**, Mohamed R. Shehata** ^2^ **and** **Ehab M. Abdalla** ^1,*^

^1^ Chemistry Department, Faculty of Science, New Valley University, Alkharga 72511, Egypt

^2^ Chemistry Department, Faculty of Science, Cairo University, Giza 12613, Egypt

*** Correspondence: E-mail: norasaad@sci.nvu.edu.eg**

**E-mail:** [**Ehababdalla99@sci.nvu.edu.eg**](mailto:Ehababdalla99@sci.nvu.edu.eg)

**Section S1: Physical Measurements**

The characterization of CBZ and its synthesized complexes of La(III), Ce(III), Nd(III), and Dy(III) complexes have been performed via different spectroscopic techniques. Fourier transforms infrared measurements for the ligand and its complexes were recorded in KBr disks using a NICOLET-is50 FTIR Thermo Electron Corporation spectrophotometer in the range of 4000–400 cm^-1^ by Central lab of Faculty of Science, New Valley University. Using the Vario EL-III Elemental Analyzer (Germany), elemental analyses (C, H, N) were conducted on the ligand and its complexes by Micro Analytical Center of the Cairo University, Egypt. ^1^H, ^13^C-NMR spectra in deuterated dimethyl sulfoxide d_6_-DMSO were recorded using a 300 MHz Varian NMR spectrometer.The spectral data of 10^-3^ M CBZ and its complexes in Dimethyl Formamide (DMF) solution were obtained utilizing 1cm quartz using a Perkin Elmer Lambada 330 spectrophotometer, the molar absorptivity (ε) (M⁻¹•cm⁻¹) is (ε)= A / (c • I) where A = measured absorbance (unitless), c = concentration (M), l = path length of cuvette (cm). The molar conductance of the ligand and its complex solutions in DMF as a solvent was determined using an Adwa AD8000 conductivity meter. Mass spectra of all new complexes were performed by Electron ionization mass spectroscopy (EI-MS) at 70 eV on the Thermo Scientific Italy-USA 2009 at the National Center for Research, Cairo. XRD data of the CBZ and its complexes were collected with a D8 Advance with DAVINCI design (Bruker, Germany), using as X-ray source the Cu Kα radiation (wavelength λ = 1.5418 Å), at 40 kV and 40 mA, a 2θ range of 20–80°, a step size of 0.02°, and a time/step of 0.6 s. A Si zero-background sample holder was used, operated by DIFFRAC. Measurements Center Version V7.3.0 (32Bit) software, while the assignment of peaks was based on the Powder Diffraction Files (PDF) of the COD database (Crystallography Open Database) in X-ray Unit, Faculty of Science, Sohag University. Furthermore, the thermograms of the complexes in air were recorded using a Thermo SDT Q600 differential thermal gravimetric analyzer at a heating rate of 20 ^o^C/min from ambient temperature to 800 ^o^C in Central lab of Faculty of Science, New Valley University. Antimicrobial and anticancer activities of ligand (CBZ) and its synthesized complexes were studied against different microorganisms and two human cancer cell lines using a modified well diffusion method and MTT assay, respectively, in Fungi Center, Al-Azhar University.

Surface morphological study was recorded by taking scanning electron microscopy (SEM) and Transmission electron microscope (TEM) images for CBZ ligand and La(III) CBZ complex in ,

Jeol ECA 500 II, 500 [MHz], 11.75 [Tesla], Made by Jeol, Japan.

**Section S2: Antimicrobial bioassay**

Briefly, 100 μl of the test bacteria/fungi were grown in 10 mL of fresh media until they reached a count of approximately 108 cells/ml for bacteria or 105 cells/mL for fungi. One hundred μl of microbial suspension was spread onto agar plates corresponding to the broth in which they were maintained and tested for susceptibility by well diffusion method. One hundred µL of each sample (at 10 mg/ml) was added to each well (6 mm diameter holes cut in the agar gel). The plates were incubated for 24-48 h at 37 °C (for bacteria and yeast) and for 48 h at 28 °C (for filamentous fungi). After incubation, the microorganism's growth was observed. The resulting inhibition zone diameters were measured in millimeters and used as criterion for the antimicrobial activity. If an organism is placed on the agar, it will not grow in the area around the well if it is susceptible to the chemical. This area of no growth around the disc is known as a "Zone of inhibition" or "Clear zone". The size of the clear zone is proportional to the inhibitory action of the compound under investigation. Solvent controls (DMSO) were included in every experiment as negative controls. DMSO was used for dissolving the tested compounds and showed no inhibition zones, confirming that it has no influence on growth of the tested microorganisms.

**Section S3: Cytotoxicity investigation**

For antitumor assays, the tumor cell lines were suspended in medium at concentration 1x10^5^ cell/well in Corning® 96-well tissue culture plates, then incubated for 24 hr. The tested compounds were dissolved in PBS then added into 96-well plates (three replicates) to achieve eight for each tested compound (twelve concentrations for Cisplatin Reference Standard). Six vehicle controls with media were run for each 96 well plate as a control. After incubating for 24 h, the numbers of viable cells were determined by the MTT test. Briefly, the media was removed from the 96 well plate and replaced with 100 µl of fresh culture RPMI 1640 medium without phenol red then 10 µl of the 12 mM MTT stock solution (5 mg of MTT in 1 mL of PBS) to each well including the untreated controls. The 96 well plates were then incubated at 37°C and 5% CO_2_ for 4 hours. An 85 µl aliquot of the media was removed from the wells, and 50 µl of DMSO was added to each well and mixed thoroughly with the pipette and incubated at 37°C for 10 min. Then, the optical density was measured at 590 nm with the microplate reader (SunRise, TECAN, Inc, USA) to determine the number of viable cells and the percentage of viability was calculated as [(ODt/ODc)]x100% where ODt is the mean optical density of wells treated with the tested sample and ODc is the mean optical density of untreated cells. The relation between surviving cells and drug concentration is plotted to get the survival curve of each tumor cell line after treatment with the specified compound. The 50% inhibitory concentration (IC_50_), the concentration required to cause toxic effects in 50% of intact cells, was estimated from graphic plots of the dose response curve for each conc. using Graphpad Prism software (San Diego, CA. USA).

**Table S1** Data of antibacterial and antifungal activities of ligand (CBZ) and its lanthanide complexes against different pathogenic microorganisms at concentration 10 mg/ml.

| **Sample**  **Tested microorganisms** | CBZ | **La(III) complex** | **Ce(III) complex** | **Nd(III) complex** | **Dy(III) complex** | **Control** |
| --- | --- | --- | --- | --- | --- | --- |
| **Fungi** |  | | | | | **Ketoconazole** |
| Aspergillus flavus  (RCMB 002002) | *NA | 17 | 13 | 15 | 12 | 16 |
| Candida albicans  RCMB 005003 (1) ATCC 10231 | NA | 15 | 12 | 13 | 11 | 20 |
| **Gram positive bacteria:** |  | | | | | **Gentamycin** |
| Staphylococcus aureus  (ATCC 25923) | 9 | 18 | 11 | 16 | 10 | 24 |
| Bacillus subtilis  RCMB 015 (1) NRRL B-543 | NA | 13 | 10 | 12 | 9 | 26 |
| **Gram negatvie bacteria:** |  | | | | | **Gentamycin** |
| Escherichia coli  (ATCC 25922) | NA | NA | NA | NA | NA | 30 |
| Proteus vulgaris  RCMB 004 (1) ATCC 13315 | 11 | 15 | 13 | 14 | 12 | 25 |

*NA: No activity.

**Table S2** Cytotoxic activity (IC_50_) of ligand (CBZ) and its lanthanide complexes against HepG2 and MCF7 Cell lines.

| **Compound** | **IC_50_ (µg/ml)** | | **IC_50_ (µM)** | |
| --- | --- | --- | --- | --- |
|  | **HepG2** | **MCF7** | **HepG2** | **MCF7** |
| **CBZ** | 59.32 | 72.49 | 0.251 | 0.307 |
| **La (III)-CBZ** | 71.82 | 82.11 | 0.095 | 0.110 |
| **Ce (III)-CBZ** | 112.10 | 145.46 | 0.142 | 0.184 |
| **Nd (III)-CBZ** | 93.65 | 120.88 | 0.120 | 0.156 |
| **Dy (III)-CBZ** | 188.40 | 231.67 | 0.242 | 0.298 |
| **Cisplatin** | 3.85 | 11.95 | 0.013 | 0.039 |

**Table S3** The Docking interaction data calculations of CBZ, [La(CBZ**)**_2_(H_2_O)Cl]^2+^, [Ce(CBZ**)**_2_(H_2_O)Cl]^2+^, [Nd(CBZ**)**_2_(H_2_O)Cl]^2+^, and [Dy(CBZ**)**_2_(H_2_O)Cl]^2+^ with the active sites of the receptor of breast cancer MCF-7 human (PDB ID: 4zvm).

|  | **Receptor** | **Interaction** | **Distance(Å)*** | **E (kcal/mol)** | S score | RMSD |
| --- | --- | --- | --- | --- | --- | --- |
| CBZ | | | | |  |  |
| O 5 | N GLY 68 (A) | H-acceptor | 3.12 (2.31) | -2.4 | -4.637 | 1.274 |
| [La(CBZ**)**_2_(H_2_O)Cl]^2+^ | | | | |  |  |
| N 43 | OE1 GLU 193 (B) | Ionic | 3.02 | -9.4 |  |  |
| N 43 | OE2 GLU 193 (B) | Ionic | 3.27 | -4.9 | -10.349 | 1.178 |
| N 45 | OE2 GLU 193 (B) | Ionic | 3.79 | -1.0 |  |  |
| 6-ring | CA GLY 68 (A) | pi-H | 3.73 | -0.5 |  |  |
| [Ce(CBZ**)**_2_(H_2_O)Cl]^2+^ | | | | |  |  |
| O 62 | NZ LYS 4 (A) | H-acceptor | 2.69 (1.92) | -5.5 | -6.850 | 1.422 |
| CE 60 | NZ LYS 4 (A) | Ionic | 2.66 | -7.2 |  |  |
| [Nd(CBZ**)**_2_(H_2_O)Cl]^2+^ | | | | |  |  |
| N 43 | OD1 ASN 66 (B) | H-donor | 2.95 (1.98) | -6.9 |  |  |
| O 61 | OE1 GLU 193 (A) | H-donor | 3.51 (2.62) | -0.8 |  |  |
| N 43 | OE2 GLU 193 (A) | Ionic | 3.22 | -3.1 | -8.292 | 1.225 |
| N 45 | OE2 GLU 193 (A) | Ionic | 3.57 | -1.7 |  |  |
| 6-ring | NE2 HIS 11 (A) | Ionic | 3.42 | -0.6 |  |  |
| 7-ring | NE2 HIS 11 (A) | pi-H | 4.57 | -0.6 |  |  |
| [Dy(CBZ**)**_2_(H_2_O)Cl]^2+^ | | | | |  |  |
| O 61 | N ASN 18 (A) | H-acceptor | 2.82 (1.97) | -7.2 |  |  |
| O 61 | ND2 ASN 18 (A) | H-acceptor | 3.18 (2.34) | -3.3 | -5.815 | 1.348 |
| N 1 | OE2 GLU 193 (A) | Ionic | 3.79 | -0.8 |  |  |

*The lengths of H-bonds are in brackets.


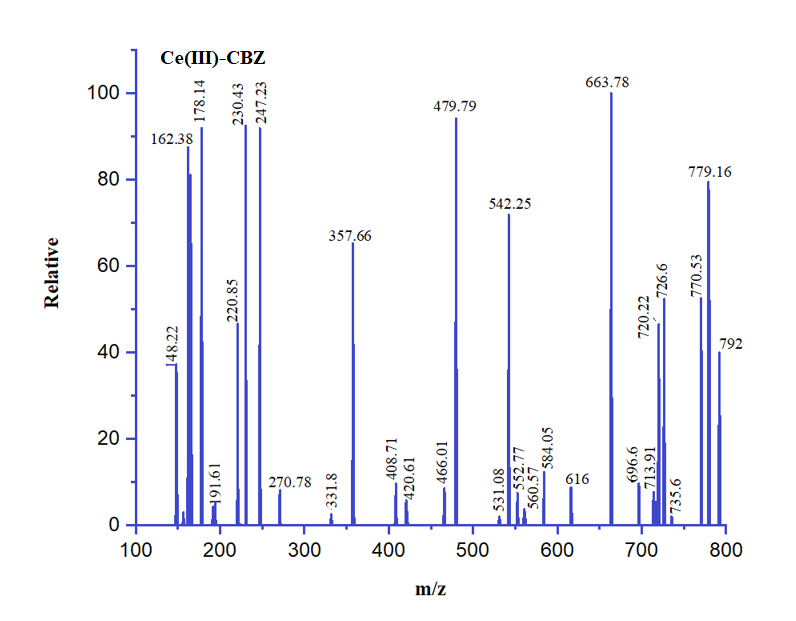


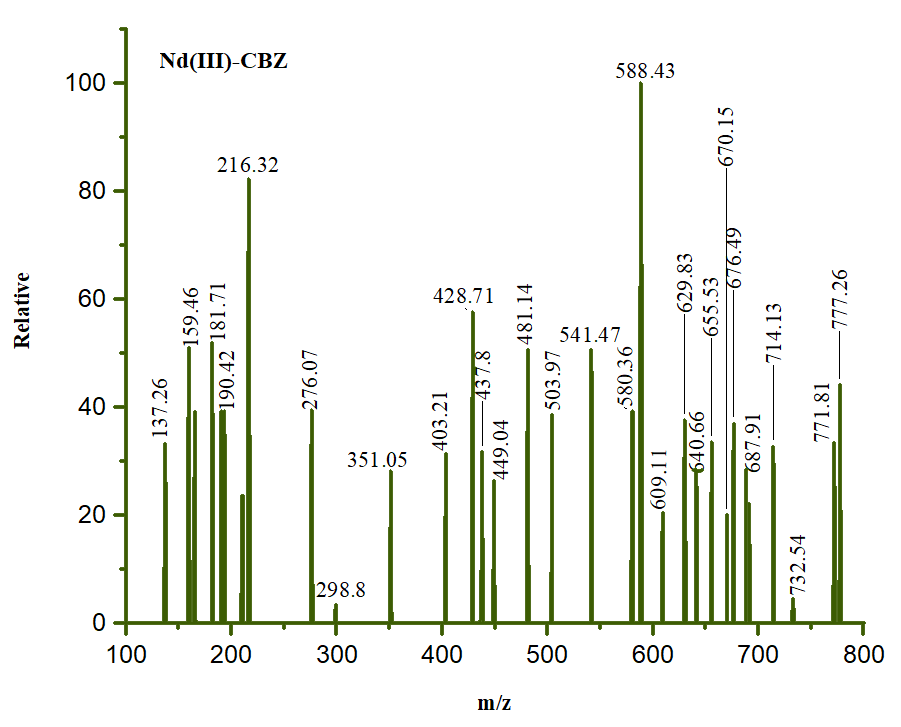


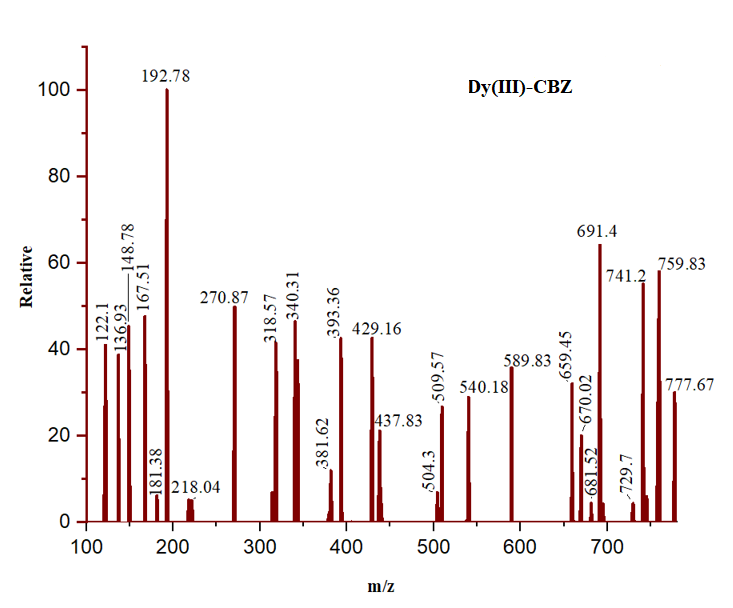


**Fig. S1.** Mass spectra diagram of Ce(III), Nd(III), Dy(III) complexes

Scheme S1 Fragmentation pattern of complex Ce(III), Nd(III) and Dy(III) complexes


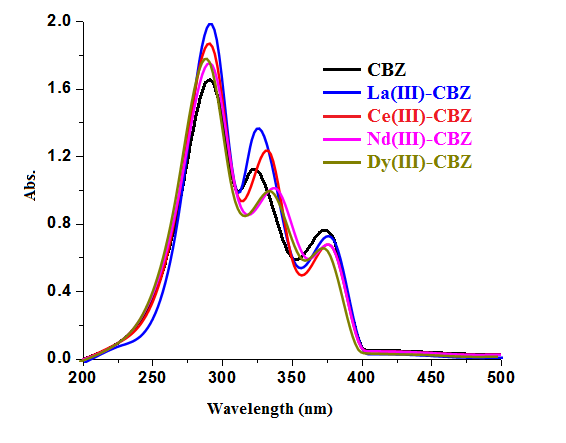


**Fig. S2.** Electronic spectra of 10^-3^ M CBZ ligand and Ln(III) complexes in DMF solution at 298 K

| **CBZ** |  | 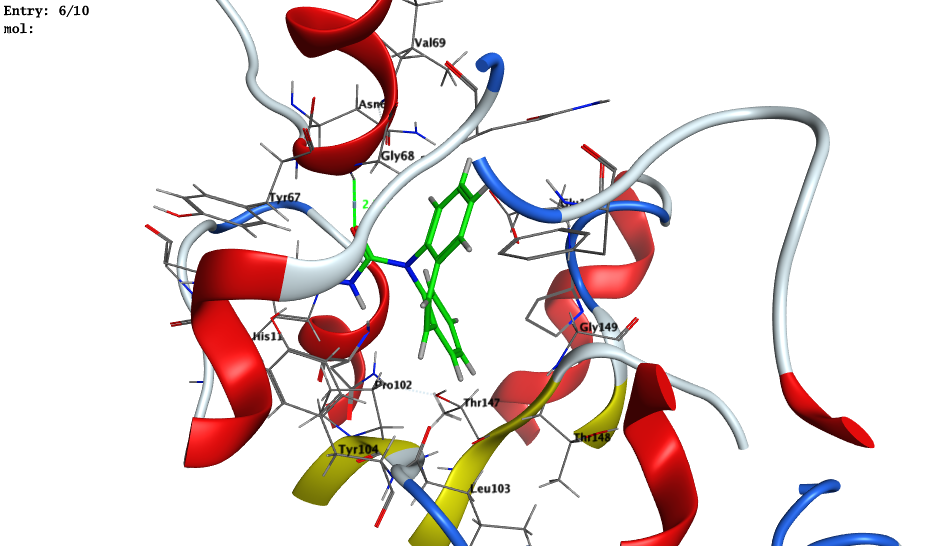 | 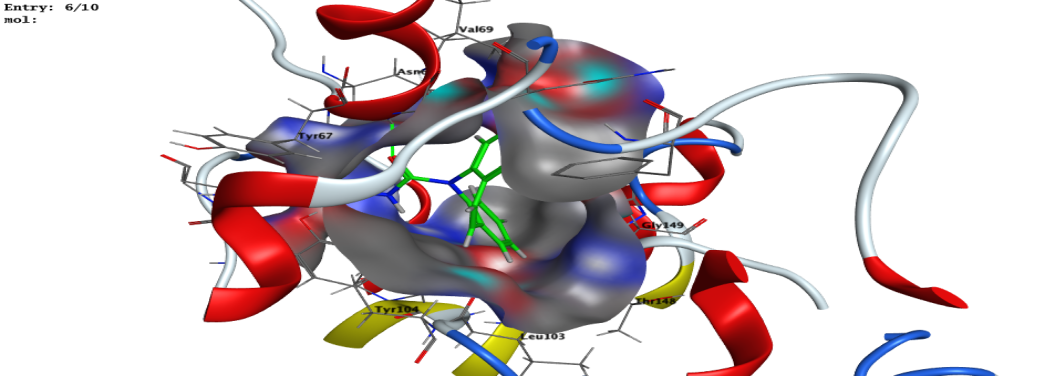 |
| --- | --- | --- | --- |
| **[La(CBZ)_2_(H_2_O)Cl]^2+^** |  | 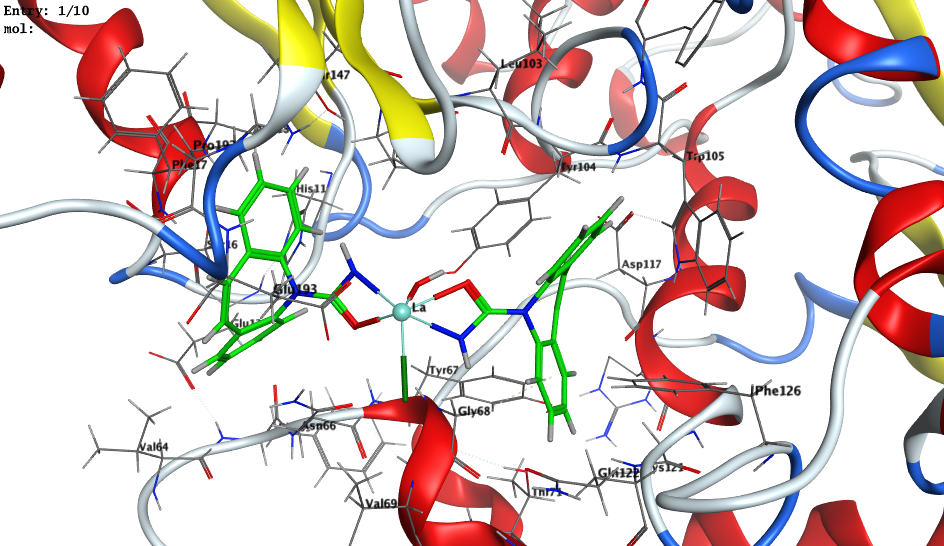 | 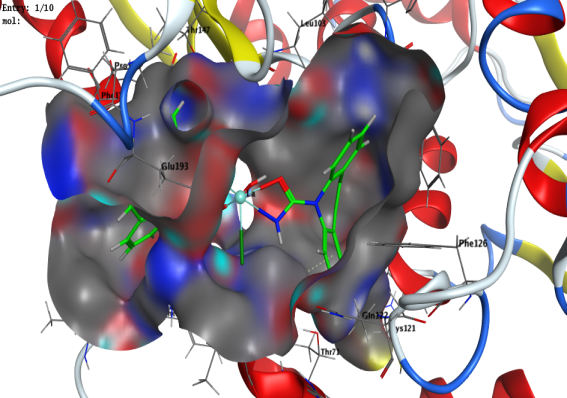 |
| **[Ce(CBZ)_2_(H_2_O)Cl]^2+^** |  | 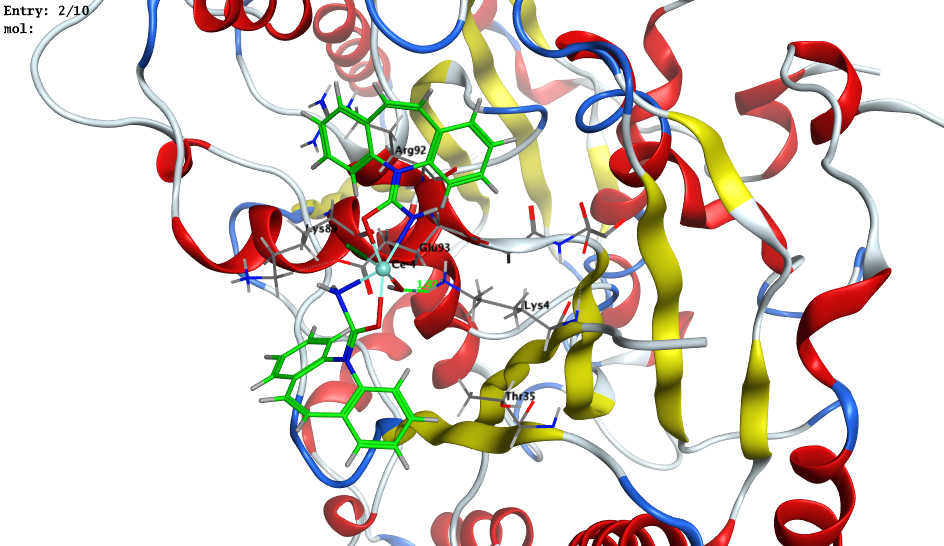 | 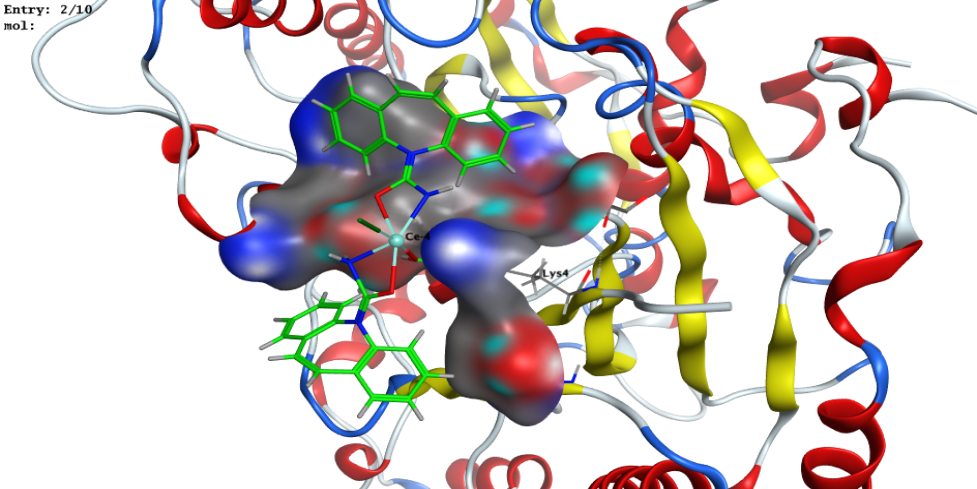 |
| **[Nd(CBZ)_2_(H_2_O)Cl]^2+^** |  | 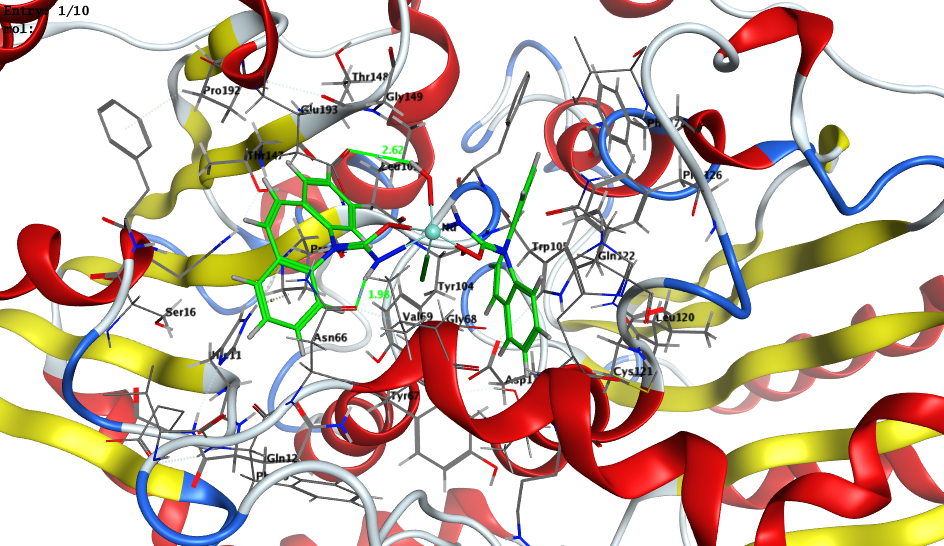 | 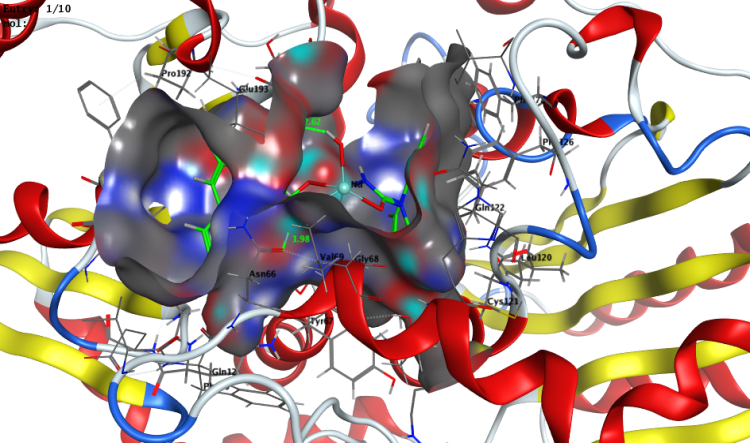 |
| **[Dy(CBZ)_2_(H_2_O)Cl]^2+^** |  | 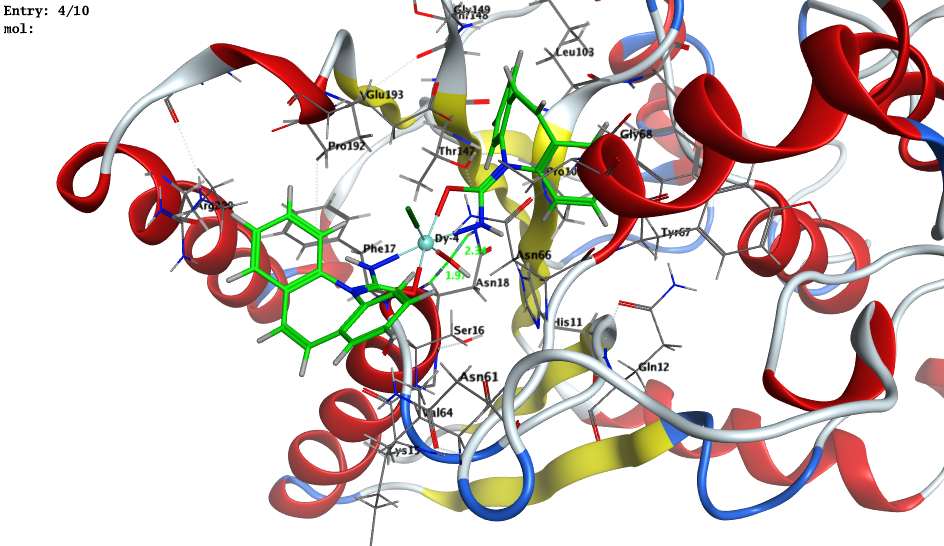 | 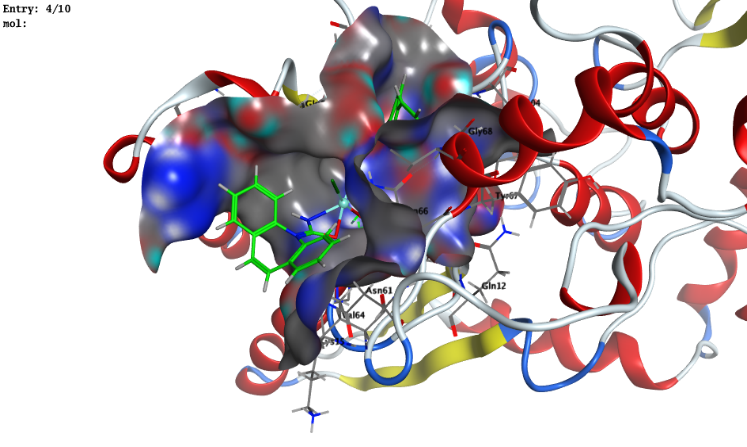 |

**Fig. S3.** 2D and 3D plots of the interaction between CBZ, [La(CBZ**)**_2_(H_2_O)Cl]^2+^, [Ce(CBZ**)**_2_(H_2_O)Cl]^2+^, [Nd(CBZ**)**_2_(H_2_O)Cl]^2+^, and [Dy(CBZ**)**_2_(H_2_O)Cl]^2+^ with the active site of the receptor of the breast cancer MCF-7 human (PDB ID: 4zvm). Hydrophobic interactions with amino acid residues are shown with dotted curves.
